# Supplementary material for: Latent brain subtypes of chronotype reveal unique behavioral and health profiles across population cohorts
Source: Nat Commun. 2025 Dec 22;16:11550. doi: 10.1038/s41467-025-66784-8 (PMC12749713; doi:10.1038/s41467-025-66784-8)
Supplement: Supplementary file 1 — Supplementary Information [file 41467_2025_66784_MOESM1_ESM.pdf]

Supplementary Information

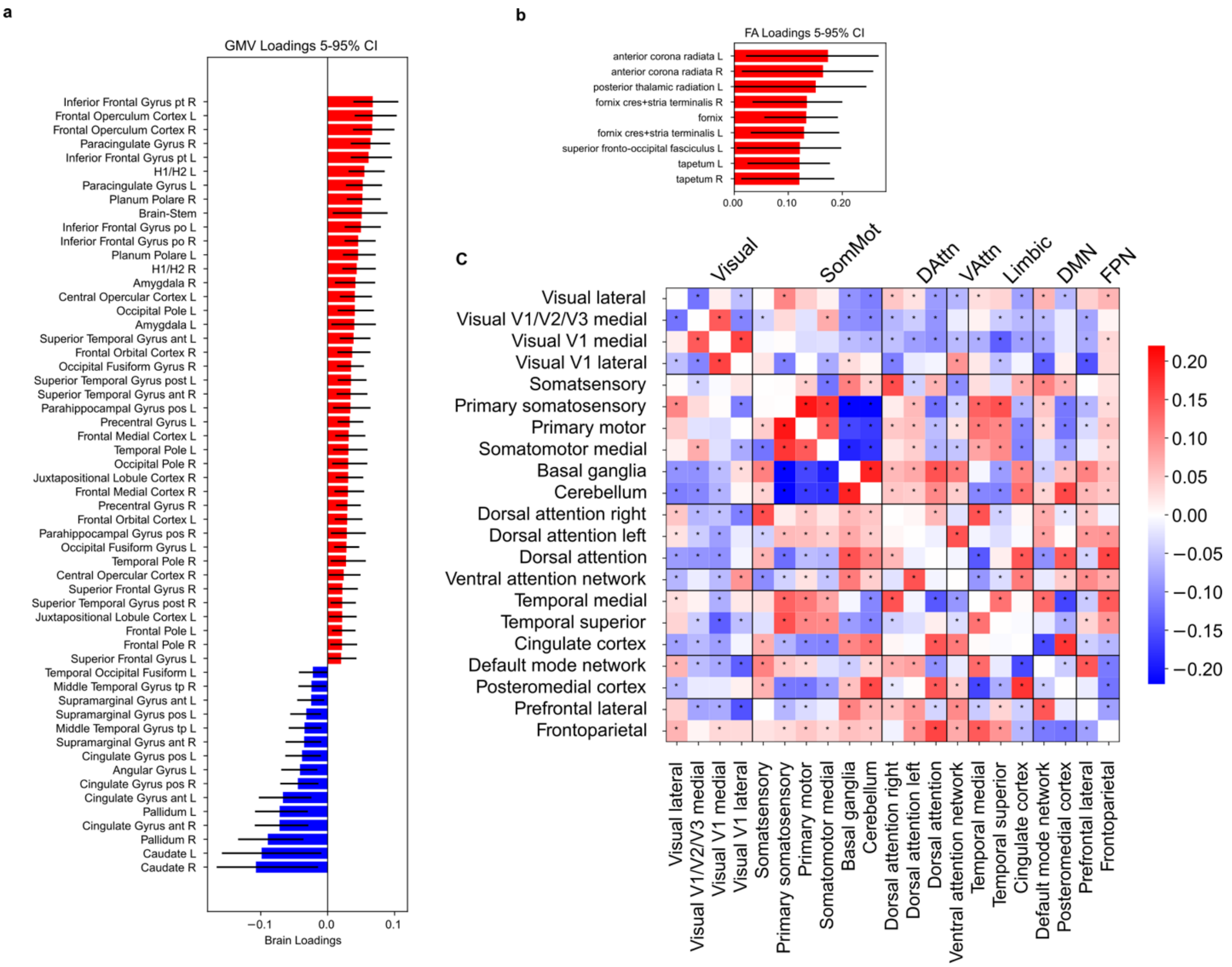

Supplementary Fig. 1 | Significant brain features derived from 1000 bootstrap iterations with 5-95% confidence interval. **a)** Significant GMV loadings. **b)** Significant FA loadings. **c)** Resting-state fMRI loadings, with significant loadings indicated by asterisk (*). Abbreviations: Visual: Visual Network; SomMot: Somatomotor Network; DAttn: Dorsal Attention Network; VAttn: Ventral Attention Network; Limbic: Limbic Network; DMN: Default Mode Network; FPN: Frontoparietal Network. Source data are provided as a Source Data file.

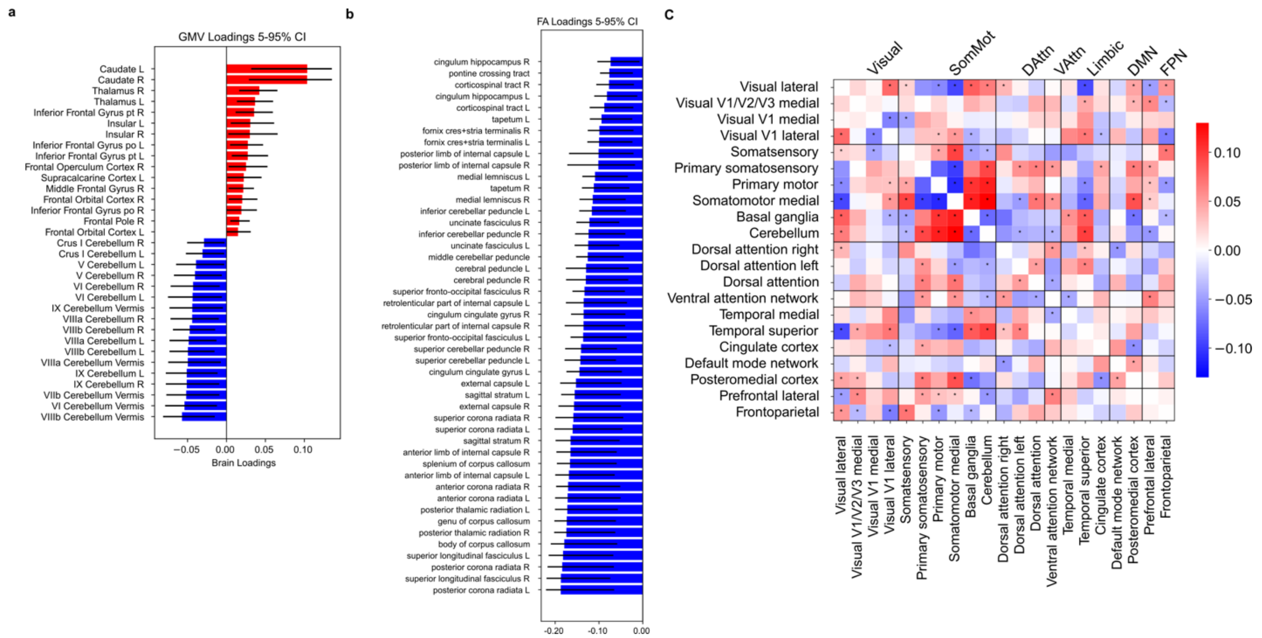

Supplementary Fig. 2 | Significant brain features derived from 1000 bootstrap iterations with 5-95% confidence interval. **a)** Significant GMV loadings. **b)** Significant FA loadings. **c)** Resting-state fMRI loadings, with significant loadings indicated by asterisk (*). Abbreviations: Visual: Visual Network; SomMot: Somatomotor Network; DAttn: Dorsal Attention Network; VAttn: Ventral Attention Network; Limbic: Limbic Network; DMN: Default Mode Network; FPN: Frontoparietal Network. Source data are provided as a Source Data file.

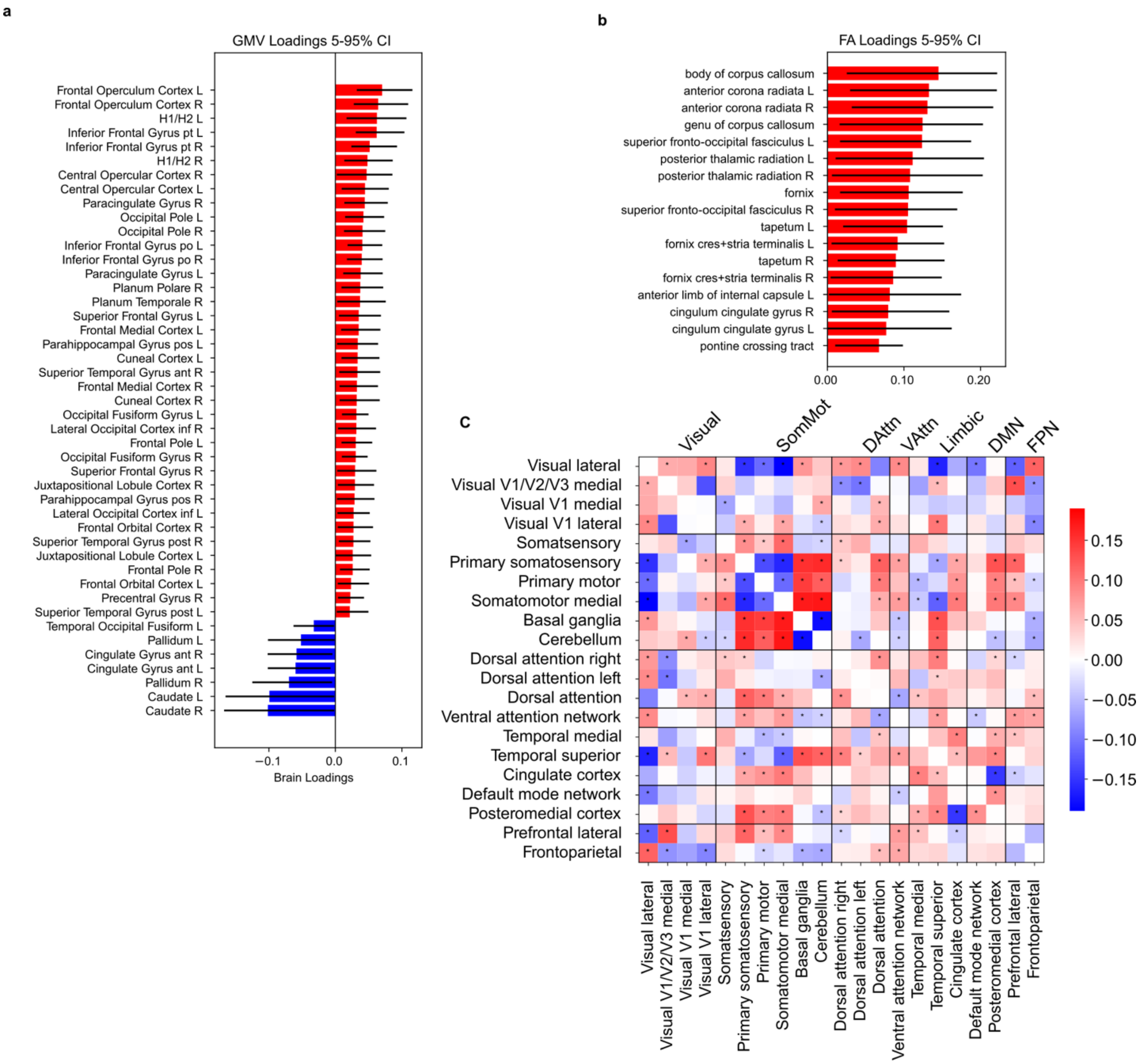

Supplementary Fig. 3 | Significant brain features derived from 1000 bootstrap iterations with 5-95% confidence interval. **a)** Significant GMV loadings. **b)** Significant FA loadings. **c)** Resting-state fMRI loadings, with significant loadings indicated by asterisk (*). Abbreviations: Visual: Visual Network; SomMot: Somatomotor Network; DAttn: Dorsal Attention Network; VAttn: Ventral Attention Network; Limbic: Limbic Network; DMN: Default Mode Network; FPN: Frontoparietal Network. Source data are provided as a Source Data file.

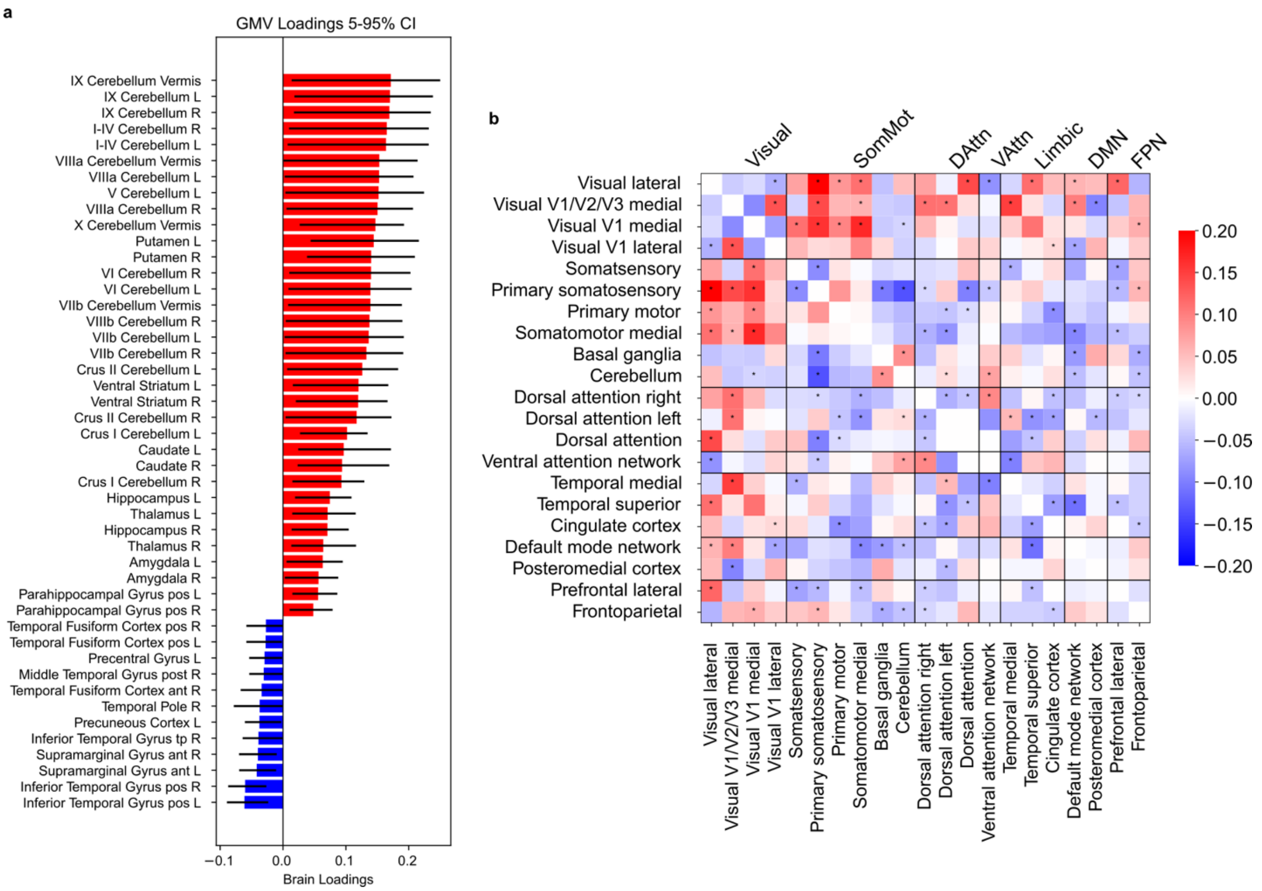

Supplementary Fig. 4 | Significant brain features derived from 1000 bootstrap iterations with 5-95% confidence interval. **a)** Significant GMV loadings. **b)** Resting-state fMRI loadings, with significant loadings indicated by asterisk (*). Abbreviations: Visual: Visual Network; SomMot: Somatomotor Network; DAttn: Dorsal Attention Network; VAttn: Ventral Attention Network; Limbic: Limbic Network; DMN: Default Mode Network; FPN: Frontoparietal Network. Source data are provided as a Source Data file.

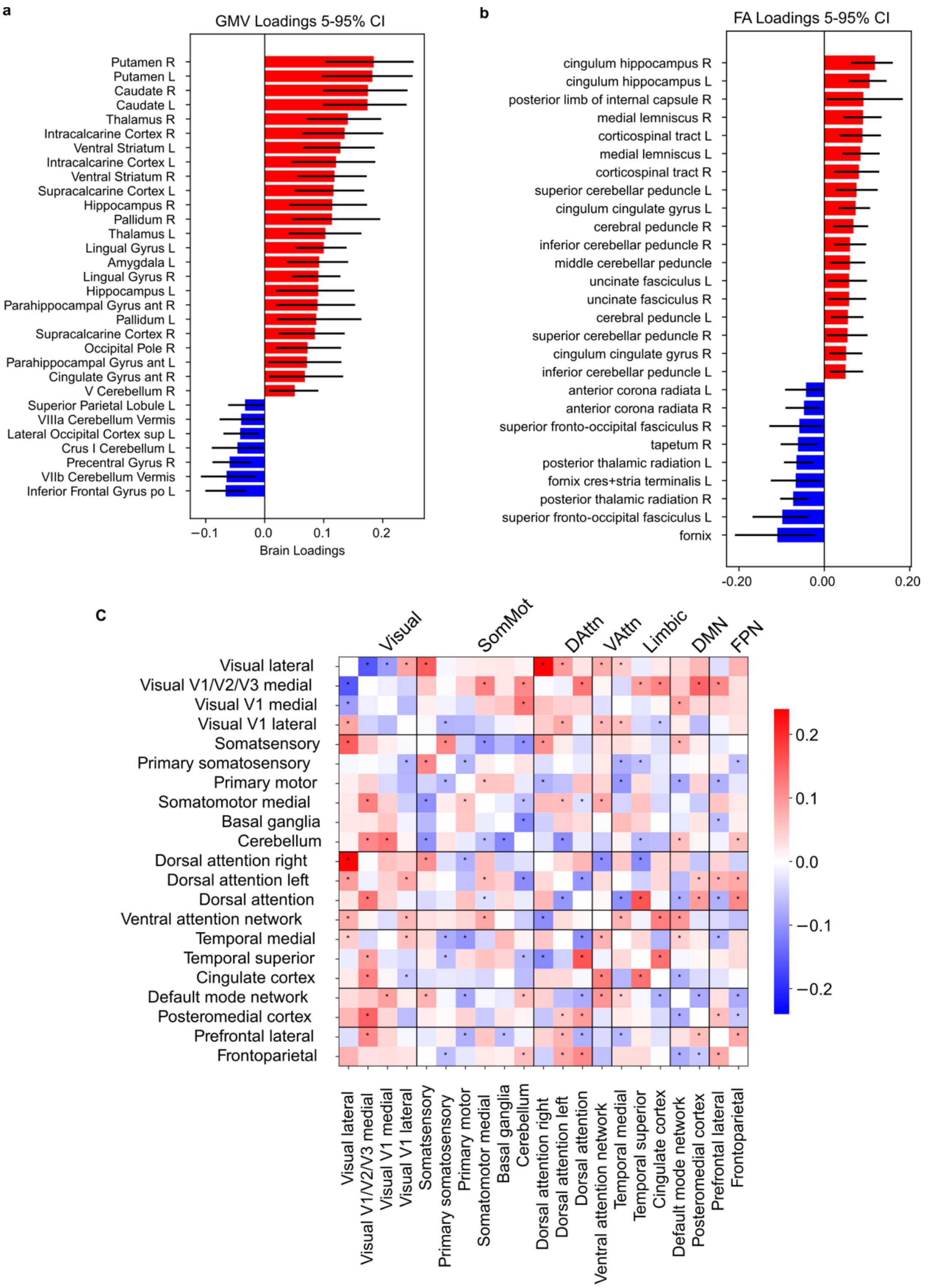

Supplementary Fig. 5 | Significant brain features derived from 1000 bootstrap iterations with 5-95% confidence interval. **a)** Significant GMV loadings. **b)** Significant FA loadings. **c)** Resting-state fMRI loadings, with significant loadings indicated by asterisk (*). Abbreviations: Visual: Visual Network; SomMot: Somatomotor Network; DAttn: Dorsal Attention Network; VAttn:

Ventral Attention Network; Limbic: Limbic Network; DMN: Default Mode Network; FPN: Frontoparietal Network. Source data are provided as a Source Data file.

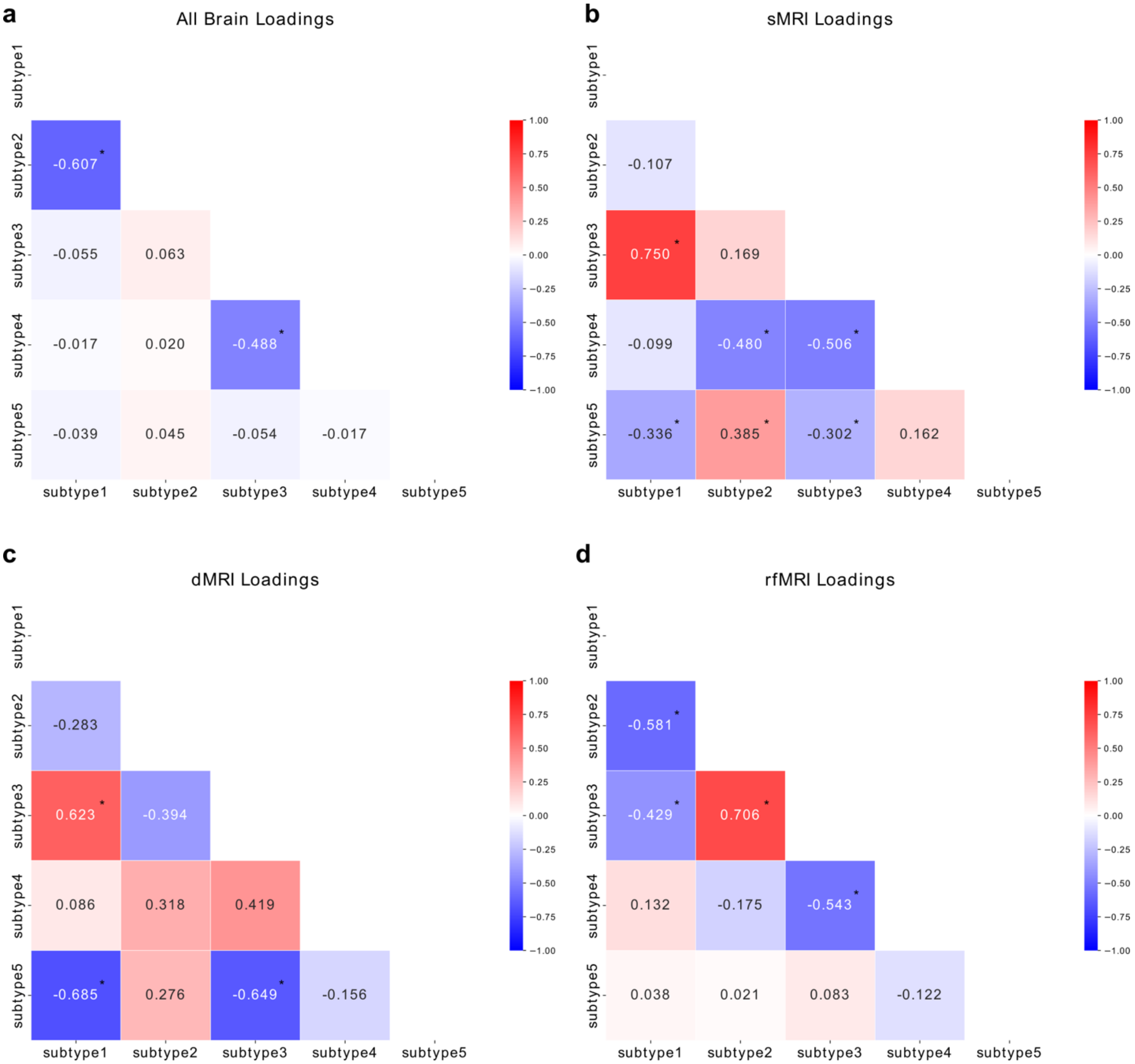

Supplementary Fig. 6 | Similarity among five brain patterns. Pearson correlation coefficients were computed between each pair of loadings and are annotated in each square. **a)** similarity of all brain loadings. **b)** similarity of GMV loadings. **c)** similarity of FA loadings. **d)** similarity of resting-state functional coupling loadings. Significant similarities are indicated by * at threshold $P < 0.001$.

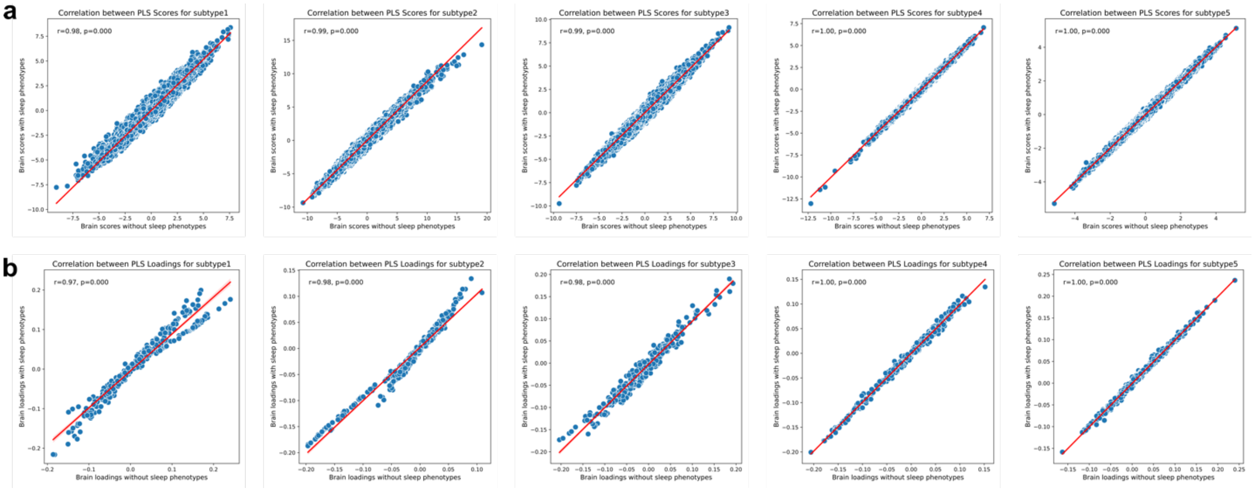

Supplementary Fig. 7 | Sensitivity analyses assessing the impact of sleep phenotypes on our main modeling. Comparison of brain scores (**a**) and brain loadings (**b**) from models with versus without adjustment for sleep phenotypes (sleep duration, insomnia, dozing, snoring, napping). Components were aligned to the five significant subtypes from the primary analysis. Pearson's correlations for all subtypes were > 0.9 (P < 0.001), indicating that adjusting for sleep phenotypes had a negligible effect on the derived patterns. Source data are provided as a Source Data file.

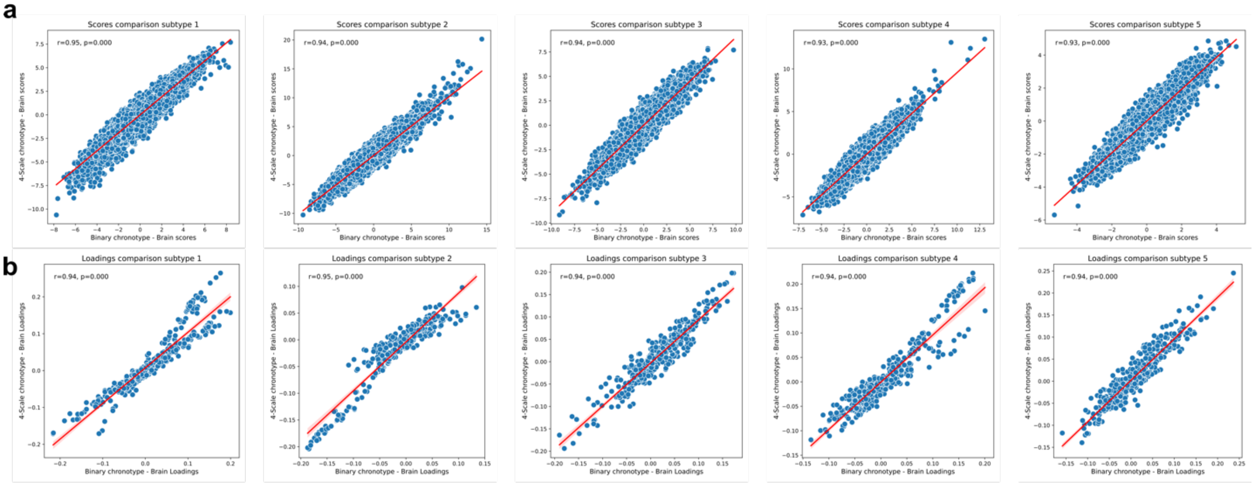

Supplementary Fig. 8 | Sensitivity analyses assessing the impact of binary chronotype on our main modeling. Comparison of brain scores (**a**) and brain loadings (**b**) from models with 4-scale versus binary scale of chronotype. Components were aligned to the five significant subtypes from the primary analysis. Pearson's correlations for all subtypes were > 0.9 (P< 0.001), indicating that binary simplification of chronotype had a negligible effect on the derived patterns. Source data are provided as a Source Data file.

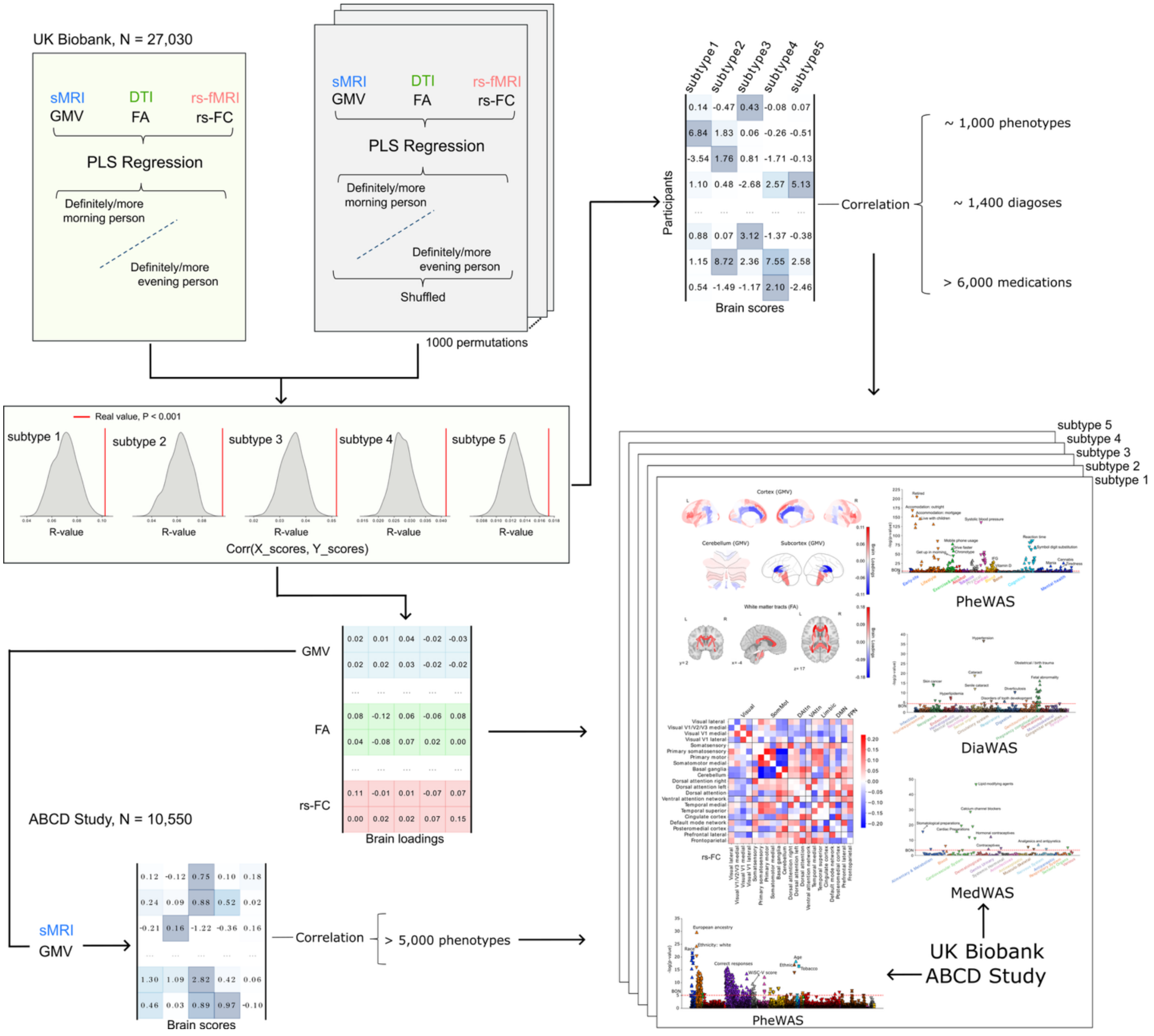

Supplementary Fig. 9 | The overall workflow of the current study. For our UK Biobank sample, the input variable set was constructed from the concatenated brain features, including 139 regional gray matter volume measures, 48 major white matter microstructure measures, and 210 functional coupling measures between 21 brain components, derived from 27,030 participants. The target variable set consisted of the dichotomized chronotype. The resulting dominant PLS components were evaluated for statistical robustness through a non-parametric permutation procedure. Specifically, in our study, the brain features were held constant while each row of the chronotype variable randomly shuffled, thus mixing group assignment between participants. This procedure generated a valid empirical null distribution of Pearson's correlation in the derived embedding space of the PLS model. It reflected the null hypothesis of a random association between brain features and chronotype. Pearson's correlation coefficients (rho) estimated between the perturbed low-rank projections were recorded in each iteration. P-values were derived based on the comparison of the original obtained rho and the 1000 rho estimates from the null PLS model, using the threshold $P < 0.001$. Five significant components (subtypes) were ultimately derived. The brain scores of the 5 subtypes were used to conduct the PheWAS, DiaWAS, and MedWAS by

correlating them with other measurements. The derived brain loadings and weights were then applied to transform GMV data from the ABCD Study® cohort into the brain scores for the children (see Methods). A subsequent PheWAS correlated these scores with other phenotypes for the children. In summary, for each subtype, we identified unique brain patterns, three-WAS results for UK Biobank participants, and PheWAS results for ABCD Study® children.

Supplementary Table 1. Demographics and sleep phenotypes of analyzed UK Biobank sample

	Morningness (coded as -1)	Eveningness (coded as 1)
N	17995	9035
Male	8128 (45.17%)	4187 (46.34%)
Female	9867 (54.83%)	4848 (53.66%)
Average Age (years)	55.71 (SD = 7.27)	54.71 (SD = 7.56)
Average Sleep duration (hours)	7.29 (SD = 1.01)	7.22 (SD = 1.03)
Nap during day (1-Never, 2-Sometimes, 3-usually)	1.49 (SD = 0.61)	1.45 (SD = 0.59)
Sleeplessness (1-Never, 2-Sometimes, 3-Usually)	2.10 (SD = 0.72)	2.08 (SD = 0.73)
Snoring (1-Yes, 2-No)	1.65 (SD = 0.48)	1.62 (SD = 0.48)
Daytime dozing (0-Never, 1-Sometimes, 2-Often, 3-All the time)	0.26 (SD = 0.48)	0.24 (SD = 0.48)

Summary of sex, average age, and average sleep phenotypes by chronotype categories. The numbers in parentheses for male and female indicate the proportions within each chronotype, while the numbers in parentheses for age and sleep phenotypes represent the standard deviations.

Supplementary Table 2. Phenotypic associations of the UK Biobank cohort

Subtype 1				
PheWAS				
**cat_id**	**phe_name**	**r_value**	**p_value**	**-logp_value**
lifestyle and environment - general	680_1.0-Own or rent accommodation lived in (0.0)	-0.167	2.769E-168	167.558
lifestyle and environment - general	680_2.0-Own or rent accommodation lived in (0.0)	0.160	4.600E-155	154.337
lifestyle and environment - general	680_4.0-Own or rent accommodation lived in (0.0)	0.027	9.535E-06	5.021
lifestyle and environment - general	2395_1.0-Hair/balding pattern (0.0)	0.025	4.240E-05	4.373
lifestyle and environment - general	1797-Father still alive (0.0)	0.143	3.884E-123	122.411
lifestyle and environment - general	1835-Mother still alive (0.0)	0.148	8.891E-133	132.051
lifestyle and environment - general	6138#3-Qualifications (0.0)	0.033	5.087E-08	7.294
lifestyle and environment - general	6138#2-Qualifications (0.0)	0.026	1.945E-05	4.711
lifestyle and environment - general	6142#1-Current employment status (0.0)	0.160	3.169E-155	154.499
lifestyle and environment - general	6142#2-Current employment status (0.0)	-0.184	2.498E-204	203.602
lifestyle and environment - general	6142#7-Current employment status (0.0)	0.030	6.104E-07	6.214
lifestyle and environment - general	6139#1-Gas or solid-fuel cooking/heating (0.0)	0.036	3.793E-09	8.421

lifestyle and environment - general	6141#2-How are people in household related to participant (0.0)	0.156	4.183E-147	146.379
lifestyle and environment - general	6145#100-Illness, injury, bereavement, stress in last 2 years (0.0)	-0.055	1.929E-19	18.715
lifestyle and environment - general	6145#5-Illness, injury, bereavement, stress in last 2 years (0.0)	0.030	7.375E-07	6.132
lifestyle and environment - general	6145#6-Illness, injury, bereavement, stress in last 2 years (0.0)	0.070	6.403E-31	30.194
lifestyle and environment - general	6145#2-Illness, injury, bereavement, stress in last 2 years (0.0)	0.033	4.696E-08	7.328
lifestyle and environment - general	20107#8-Illnesses of father (0.0)	0.028	4.526E-06	5.344
lifestyle and environment - general	20107#9-Illnesses of father (0.0)	0.033	8.036E-08	7.095
lifestyle and environment - general	20110#100-Illnesses of mother (0.0)	0.036	2.670E-09	8.573
lifestyle and environment - general	20110#2-Illnesses of mother (0.0)	-0.043	8.672E-13	12.062
lifestyle and environment - general	20110#10-Illnesses of mother (0.0)	-0.037	1.364E-09	8.865
lifestyle and environment - general	20111#100-Illnesses of siblings (0.0)	0.043	2.089E-12	11.680
lifestyle and environment - general	20111#8-Illnesses of siblings (0.0)	-0.040	4.375E-11	10.359
lifestyle and environment - general	709-Number in household (0.0)	0.148	4.047E-132	131.393
lifestyle and environment - general	728-Number of vehicles in household (0.0)	0.046	3.983E-14	13.400
lifestyle and environment - general	738-Average total household income before tax (0.0)	0.086	6.360E-46	45.197
lifestyle and environment - general	777-Frequency of travelling from home to job workplace (0.0)	0.033	8.156E-08	7.089
lifestyle and environment - general	1031-Frequency of friend/family visits (0.0)	0.044	8.344E-13	12.079
lifestyle and environment - general	1873-Number of full brothers (0.0)	0.040	4.823E-11	10.317
lifestyle and environment - general	1883-Number of full sisters (0.0)	0.032	1.243E-07	6.905
lifestyle and environment - general	2149-Lifetime number of sexual partners (0.0)	0.081	7.320E-41	40.135
lifestyle and environment - general	2237-Plays computer games (0.0)	0.045	7.461E-14	13.127
lifestyle and environment - general	189-Townsend deprivation index at recruitment (0.0)	0.045	9.031E-14	13.044
lifestyle and environment - general	699-Length of time at current address (0.0)	-0.118	4.221E-85	84.375
lifestyle and environment - general	1807-Father's age at death (0.0)	-0.030	5.677E-07	6.246
lifestyle and environment - general	1845-Mother's age (0.0)	-0.103	4.697E-65	64.328
lifestyle and environment - general	2139-Age first had sexual intercourse (0.0)	-0.076	1.453E-35	34.838
lifestyle and environment - general	2946-Father's age (0.0)	-0.045	1.842E-13	12.735
lifestyle and environment - general	3526-Mother's age at death (0.0)	-0.044	5.588E-13	12.253
lifestyle and environment - general	24003-Nitrogen dioxide air pollution; 2010 (0.0)	0.033	3.612E-08	7.442
lifestyle and environment - general	24004-Nitrogen oxides air pollution; 2010 (0.0)	0.032	1.547E-07	6.810

lifestyle and environment - general	24006-Particulate matter air pollution (pm2.5); 2010 (0.0)	0.026	2.184E-05	4.661
lifestyle and environment - general	24016-Nitrogen dioxide air pollution; 2005 (0.0)	0.030	1.106E-06	5.956
lifestyle and environment - general	24017-Nitrogen dioxide air pollution; 2006 (0.0)	0.030	8.706E-07	6.060
lifestyle and environment - general	24018-Nitrogen dioxide air pollution; 2007 (0.0)	0.027	1.178E-05	4.929
lifestyle and environment - general	24506-Natural environment percentage, buffer 1000m (0.0)	-0.028	3.703E-06	5.431
lifestyle and environment - general	24507-Natural environment percentage, buffer 300m (0.0)	-0.031	4.851E-07	6.314
lifestyle and environment - general	26410-Index of Multiple Deprivation (England) (0.0)	0.030	6.600E-07	6.180
lifestyle and environment - general	26411-Income score (England) (0.0)	0.041	1.235E-11	10.908
lifestyle and environment - general	26412-Employment score (England) (0.0)	0.030	8.394E-07	6.076
lifestyle and environment - general	26414-Education score (England) (0.0)	0.029	1.541E-06	5.812
lifestyle and environment - general	26416-Crime score (England) (0.0)	0.026	2.395E-05	4.621
lifestyle and environment - general	26417-Living environment score (England) (0.0)	0.033	7.433E-08	7.129
lifestyle and environment - exercise and work	1150_2.0-Usual side of head for mobile phone use (0.0)	0.029	1.252E-06	5.902
lifestyle and environment - exercise and work	1150_3.0-Usual side of head for mobile phone use (0.0)	0.032	1.085E-07	6.965
lifestyle and environment - exercise and work	6162#1-Types of transport used (excluding work) (0.0)	0.026	1.321E-05	4.879
lifestyle and environment - exercise and work	6162#2-Types of transport used (excluding work) (0.0)	-0.036	2.499E-09	8.602
lifestyle and environment - exercise and work	6162#4-Types of transport used (excluding work) (0.0)	0.028	2.838E-06	5.547
lifestyle and environment - exercise and work	6162#3-Types of transport used (excluding work) (0.0)	-0.055	2.414E-19	18.617
lifestyle and environment - exercise and work	6164#1-Types of physical activity in last 4 weeks (0.0)	-0.042	7.934E-12	11.101
lifestyle and environment - exercise and work	6164#2-Types of physical activity in last 4 weeks (0.0)	0.026	1.437E-05	4.843
lifestyle and environment - exercise and work	6164#3-Types of physical activity in last 4 weeks (0.0)	0.067	1.927E-28	27.715
lifestyle and environment - exercise and work	826-Job involves shift work (0.0)	-0.038	3.153E-10	9.501
lifestyle and environment - exercise and work	845-Age completed full time education (0.0)	0.038	5.122E-10	9.291
lifestyle and environment - exercise and work	864-Number of days/week walked 10+ minutes (0.0)	-0.064	5.991E-26	25.223
lifestyle and environment - exercise and work	884-Number of days/week of moderate	-0.039	1.589E-10	9.799

	physical activity 10+ minutes (0.0)			
lifestyle and environment - exercise and work	924-Usual walking pace (0.0)	0.026	2.667E-05	4.574
lifestyle and environment - exercise and work	971-Frequency of walking for pleasure in last 4 weeks (0.0)	-0.040	5.749E-11	10.240
lifestyle and environment - exercise and work	981-Duration walking for pleasure (0.0)	-0.032	1.003E-07	6.998
lifestyle and environment - exercise and work	1070-Time spent watching television (TV) (0.0)	-0.045	1.563E-13	12.806
lifestyle and environment - exercise and work	1080-Time spent using computer (0.0)	0.026	1.377E-05	4.861
lifestyle and environment - exercise and work	1100-Drive faster than motorway speed limit (0.0)	0.104	1.095E-65	64.961
lifestyle and environment - exercise and work	1110-Length of mobile phone use (0.0)	0.075	1.936E-35	34.713
lifestyle and environment - exercise and work	1120-Weekly usage of mobile phone in last 3 months (0.0)	0.114	1.322E-78	77.879
lifestyle and environment - exercise and work	1130-Hands-free device/speakerphone use with mobile phone in last 3 month (0.0)	0.071	1.390E-31	30.857
lifestyle and environment - exercise and work	1140-Difference in mobile phone use compared to two years previously (0.0)	0.045	8.616E-14	13.065
lifestyle and environment - exercise and work	1160-Sleep duration (0.0)	-0.028	3.301E-06	5.481
lifestyle and environment - exercise and work	1170-Getting up in morning (0.0)	-0.086	2.676E-45	44.573
lifestyle and environment - exercise and work	1180-Morning/evening person (chronotype) (0.0)	0.099	1.040E-59	58.983
lifestyle and environment - exercise and work	1200-Sleeplessness / insomnia (0.0)	-0.040	2.850E-11	10.545
lifestyle and environment - exercise and work	2634-Duration of heavy DIY (0.0)	0.026	2.441E-05	4.612
lifestyle and environment - exercise and work	894-Duration of moderate activity (0.0)	-0.026	1.757E-05	4.755
lifestyle and environment - alcohol	2664_5.0-Reason for reducing amount of alcohol drunk (0.0)	0.051	3.396E-17	16.469
lifestyle and environment - alcohol	1558-Alcohol intake frequency. (0.0)	-0.050	1.003E-16	15.999
lifestyle and environment - alcohol	1588-Average weekly beer plus cider intake (0.0)	0.035	6.210E-09	8.207
lifestyle and environment - alcohol	1628-Alcohol intake versus 10 years previously (0.0)	0.025	3.956E-05	4.403
lifestyle and environment - alcohol	20416-Frequency of consuming six or more units of alcohol (0.0)	0.029	2.298E-06	5.639
lifestyle and environment - tobacco	2877_3.0-Type of tobacco previously smoked (0.0)	-0.025	4.564E-05	4.341

lifestyle and environment - tobacco	3446_2.0-Type of tobacco currently smoked (0.0)	0.027	7.872E-06	5.104
lifestyle and environment - tobacco	20116_1.0-Smoking status (0.0)	-0.026	1.469E-05	4.833
lifestyle and environment - tobacco	20116_2.0-Smoking status (0.0)	0.035	1.209E-08	7.918
lifestyle and environment - tobacco	1239-Current tobacco smoking (0.0)	0.035	6.258E-09	8.204
physical measures - general	46-Hand grip strength (left) (0.0)	0.068	3.388E-29	28.470
physical measures - general	47-Hand grip strength (right) (0.0)	0.063	6.495E-25	24.187
physical measures - general	3062-Forced vital capacity (FVC) (0.0)	0.060	2.935E-23	22.532
physical measures - general	3063-Forced expiratory volume in 1-second (FEV1) (0.0)	0.074	6.053E-34	33.218
physical measures - general	3064-Peak expiratory flow (PEF) (0.0)	0.073	4.621E-33	32.335
physical measures - general	12143-Weight (pre-imaging) (2.0)	0.036	2.745E-09	8.562
physical measures - general	23099-Body fat percentage (0.0)	-0.042	5.435E-12	11.265
physical measures - general	23100-Whole body fat mass (0.0)	-0.034	2.126E-08	7.672
physical measures - general	23101-Whole body fat-free mass (0.0)	0.033	5.215E-08	7.283
physical measures - general	23102-Whole body water mass (2.0)	0.049	1.461E-15	14.835
physical measures - general	23111-Leg fat percentage (right) (0.0)	-0.036	4.701E-09	8.328
physical measures - general	23113-Leg fat-free mass (right) (0.0)	0.043	2.711E-12	11.567
physical measures - general	23121-Arm fat-free mass (right) (0.0)	0.027	1.104E-05	4.957
physical measures - general	23127-Trunk fat percentage (0.0)	-0.051	6.111E-17	16.214
physical measures - general	23128-Trunk fat mass (0.0)	-0.039	1.923E-10	9.716
physical measures - cardiac & blood vessels	102-Pulse rate, automated reading (0.0)	-0.055	1.435E-19	18.843
physical measures - cardiac & blood vessels	4079-Diastolic blood pressure, automated reading (0.0)	-0.089	1.767E-48	47.753
physical measures - cardiac & blood vessels	4080-Systolic blood pressure, automated reading (0.0)	-0.150	7.708E-136	135.113
physical measures - cardiac & blood vessels	4196-Pulse wave peak to peak time (0.0)	0.029	2.520E-06	5.599
physical measures - cardiac & blood vessels	6032-Maximum workload during fitness test (0.0)	0.026	2.057E-05	4.687
physical measures - cardiac & blood vessels	12336-Ventricular rate (2.0)	-0.073	1.376E-33	32.861
physical measures - cardiac & blood vessels	22670-Minimum carotid IMT (intima-medial thickness) at 120 degrees (2.0)	-0.072	8.675E-33	32.062
physical measures - cardiac & blood vessels	22671-Mean carotid IMT (intima-medial thickness) at 120 degrees (2.0)	-0.091	3.946E-51	50.404
physical measures - cardiac & blood vessels	22672-Maximum carotid IMT (intima-medial thickness) at 120 degrees (2.0)	-0.088	1.279E-47	46.893
physical measures - cardiac & blood vessels	22673-Minimum carotid IMT (intima-medial	-0.088	4.262E-48	47.370

	thickness) at 150 degrees (2.0)			
physical measures - cardiac & blood vessels	22674-Mean carotid IMT (intima-medial thickness) at 150 degrees (2.0)	-0.095	4.332E-55	54.363
physical measures - cardiac & blood vessels	22675-Maximum carotid IMT (intima-medial thickness) at 150 degrees (2.0)	-0.087	9.169E-47	46.038
physical measures - cardiac & blood vessels	22676-Minimum carotid IMT (intima-medial thickness) at 210 degrees (2.0)	-0.071	6.571E-32	31.182
physical measures - cardiac & blood vessels	22677-Mean carotid IMT (intima-medial thickness) at 210 degrees (2.0)	-0.078	1.671E-37	36.777
physical measures - cardiac & blood vessels	22678-Maximum carotid IMT (intima-medial thickness) at 210 degrees (2.0)	-0.070	1.792E-30	29.747
physical measures - cardiac & blood vessels	22679-Minimum carotid IMT (intima-medial thickness) at 240 degrees (2.0)	-0.061	5.265E-24	23.279
physical measures - cardiac & blood vessels	22680-Mean carotid IMT (intima-medial thickness) at 240 degrees (2.0)	-0.073	5.692E-33	32.245
physical measures - cardiac & blood vessels	22681-Maximum carotid IMT (intima-medial thickness) at 240 degrees (2.0)	-0.065	6.494E-27	26.188
cognitive phenotypes	20179_2.0-FI8 : chained arithmetic (0.0)	0.026	1.517E-05	4.819
cognitive phenotypes	20246_3.0-Trail making completion status (0.0)	-0.032	1.278E-07	6.894
cognitive phenotypes	6332#5-Item selected for each puzzle (2.0)	0.029	2.492E-06	5.604
cognitive phenotypes	6332#0-Item selected for each puzzle (2.0)	0.025	3.530E-05	4.452
cognitive phenotypes	398-Number of correct matches in round (2.1)	0.065	1.549E-26	25.810
cognitive phenotypes	6351-Total errors traversing alphanumeric path (trail #2) (2.0)	-0.040	3.462E-11	10.461
cognitive phenotypes	6373-Number of puzzles correctly solved (2.0)	0.076	1.130E-35	34.947
cognitive phenotypes	6374-Number of puzzles viewed (2.0)	0.031	4.545E-07	6.342
cognitive phenotypes	6382-n/a (2.0)	0.081	1.303E-40	39.885
cognitive phenotypes	6383-Number of puzzles attempted (2.0)	0.045	7.525E-14	13.124
cognitive phenotypes	399-Number of incorrect matches in round (0.1)	-0.063	3.162E-25	24.500
cognitive phenotypes	400-Time to complete round (0.1)	-0.107	1.622E-69	68.790
cognitive phenotypes	404-Duration to first press of snap-button in each round (0.0)	-0.116	6.120E-81	80.213
cognitive phenotypes	4253-Time number displayed for (2.0)	0.047	9.829E-15	14.007
cognitive phenotypes	4290-Duration screen displayed (0.0)	-0.031	2.910E-07	6.536
cognitive phenotypes	6333-Duration spent answering each puzzle (2.0)	-0.026	1.973E-05	4.705

cognitive phenotypes	6348-Duration to complete numeric path (trail #1) (2.0)	-0.101	1.730E-62	61.762
cognitive phenotypes	6350-Duration to complete alphanumeric path (trail #2) (2.0)	-0.116	1.550E-81	80.810
cognitive phenotypes	6772-Interval between previous point and current one in numeric path (trail #1) (2.0)	-0.100	1.854E-61	60.732
cognitive phenotypes	6773-Interval between previous point and current one in alphanumeric path (trail #2) (2.0)	-0.119	7.800E-86	85.108
cognitive phenotypes	20023-Mean time to correctly identify matches (0.0)	-0.120	1.041E-86	85.983
cognitive phenotypes	20132-Number of incorrect matches in round (0.0)	-0.033	5.586E-08	7.253
cognitive phenotypes	20133-Time to complete round (0.0)	-0.069	6.702E-30	29.174
cognitive phenotypes	20149-Interval between previous point and current one in numeric path (trail #1) (0.1)	-0.062	8.665E-25	24.062
cognitive phenotypes	20153-Forced expiratory volume in 1-second (FEV1), predicted (0.0)	0.033	4.438E-08	7.353
cognitive phenotypes	20155-Interval between previous point and current one in alphanumeric path (trail #2) (0.1)	-0.072	2.821E-32	31.550
cognitive phenotypes	20156-Duration to complete numeric path (trail #1) (0.0)	-0.067	1.231E-28	27.910
cognitive phenotypes	20157-Duration to complete alphanumeric path (trail #2) (0.0)	-0.080	2.070E-39	38.684
cognitive phenotypes	20159-Number of symbol digit matches made correctly (0.0)	0.078	7.026E-38	37.153
cognitive phenotypes	20195-Number of symbol digit matches attempted (0.0)	0.077	3.055E-37	36.515
cognitive phenotypes	20230-Duration to entering value (0.0)	-0.081	2.419E-40	39.616
cognitive phenotypes	23323-Number of symbol digit matches attempted (2.0)	0.107	3.565E-70	69.448
cognitive phenotypes	23324-Number of symbol digit matches made correctly (2.0)	0.106	5.422E-68	67.266
mental health self-report	20526_1.0-Been in serious accident believed to be life-threatening (0.0)	0.025	4.610E-05	4.336
mental health self-report	20529_0.0-Victim of physically violent crime (0.0)	-0.032	1.581E-07	6.801
mental health self-report	20529_1.0-Victim of physically violent crime (0.0)	0.038	4.905E-10	9.309
mental health self-report	20531_0.0-Victim of sexual assault (0.0)	-0.026	1.548E-05	4.810
mental health self-report	20531_1.0-Victim of sexual assault (0.0)	0.035	1.160E-08	7.935

mental health self-report	20536_1.0-Weight change during worst episode of depression (0.0)	0.038	2.956E-10	9.529
mental health self-report	1960-Fed-up feelings (0.0)	0.031	2.262E-07	6.645
mental health self-report	1970-Nervous feelings (0.0)	-0.035	8.281E-09	8.082
mental health self-report	1980-Worrier / anxious feelings (0.0)	-0.039	2.012E-10	9.696
mental health self-report	2040-Risk taking (0.0)	0.043	1.435E-12	11.843
mental health self-report	20441-Ever had prolonged loss of interest in normal activities (0.0)	0.036	5.007E-09	8.300
mental health self-report	20480-Ever self-harmed (0.0)	0.028	3.427E-06	5.465
mental health self-report	20499-Ever sought or received professional help for mental distress (0.0)	0.026	2.622E-05	4.581
mental health self-report	20500-Ever suffered mental distress preventing usual activities (0.0)	0.027	6.365E-06	5.196
mental health self-report	20502-Ever had period extreme irritability (0.0)	0.054	8.135E-19	18.090
mental health self-report	20535-Waking too early (0.0)	-0.028	2.911E-06	5.536
mental health self-report	20547#1-Activities undertaken to treat depression (0.1)	0.029	2.151E-06	5.667
mental health self-report	20548#7-Manifestations of mania or irritability (0.1)	0.029	1.602E-06	5.795
mental health self-report	20548#8-Manifestations of mania or irritability (0.1)	0.033	5.484E-08	7.261
mental health self-report	20548#6-Manifestations of mania or irritability (0.1)	0.030	9.580E-07	6.019
mental health self-report	20553#4-Methods of self-harm used (0.1)	0.026	2.317E-05	4.635
mental health self-report	20553#5-Methods of self-harm used (0.1)	0.027	1.018E-05	4.992
mental health self-report	2050-Frequency of depressed mood in last 2 weeks (0.0)	0.035	1.040E-08	7.983
mental health self-report	2060-Frequency of unenthusiasm / disinterest in last 2 weeks (0.0)	0.038	3.185E-10	9.497
mental health self-report	2070-Frequency of tenseness / restlessness in last 2 weeks (0.0)	0.027	6.139E-06	5.212
mental health self-report	2080-Frequency of tiredness / lethargy in last 2 weeks (0.0)	0.051	7.434E-17	16.129
mental health self-report	4581-Financial situation satisfaction (0.0)	0.028	3.460E-06	5.461
mental health self-report	20453-Ever taken cannabis (0.0)	0.065	6.729E-27	26.172
mental health self-report	20479-Ever thought that life not worth living (0.0)	0.034	2.862E-08	7.543
mental health self-report	20485-Ever contemplated self-harm (0.0)	0.039	1.756E-10	9.756
mental health self-report	20488-Physically abused by family as a child (0.0)	0.033	4.716E-08	7.326

mental health self-report	20491-Someone to take to doctor when needed as a child (0.0)	0.033	6.674E-08	7.176
mental health self-report	20511-Recent poor appetite or overeating (0.0)	0.027	7.695E-06	5.114
mental health self-report	20519-Recent feelings of tiredness or low energy (0.0)	0.028	5.819E-06	5.235
mental health self-report	20521-Belittlement by partner or ex-partner as an adult (0.0)	0.026	1.754E-05	4.756
mental health self-report	20433-Age at first episode of depression (0.0)	-0.027	9.281E-06	5.032
mental health self-report	20434-Age at last episode of depression (0.0)	-0.045	8.468E-14	13.072
early life factors	1677-Breastfed as a baby (0.0)	-0.036	3.828E-09	8.417
early life factors	1687-Comparative body size at age 10 (0.0)	-0.033	8.081E-08	7.093
early life factors	20022-Birth weight (0.0)	-0.030	7.283E-07	6.138
physical measures - bone density and sizes	78-Heel bone mineral density (BMD) T-score, automated (0.0)	0.032	1.470E-07	6.833
physical measures - bone density and sizes	3144-Heel Broadband ultrasound attenuation, direct entry (0.0)	0.033	8.539E-08	7.069
physical measures - bone density and sizes	4101-Heel broadband ultrasound attenuation (left) (0.0)	0.027	6.562E-06	5.183
physical measures - bone density and sizes	4120-Heel broadband ultrasound attenuation (right) (0.0)	0.025	3.792E-05	4.421
physical measures - bone density and sizes	4123-Heel quantitative ultrasound index (QUI), direct entry (right) (0.0)	0.030	7.996E-07	6.097
blood assays	30040-Mean corpuscular volume (0.0)	-0.043	2.083E-12	11.681
blood assays	30050-Mean corpuscular haemoglobin (0.0)	-0.033	6.703E-08	7.174
blood assays	30100-Mean platelet (thrombocyte) volume (0.0)	0.035	5.709E-09	8.243
blood assays	30270-Mean sphered cell volume (0.0)	-0.035	6.290E-09	8.201
blood assays	30610-Alkaline phosphatase (0.0)	-0.056	2.525E-20	19.598
blood assays	30630-Apolipoprotein A (0.0)	-0.055	1.521E-19	18.818
blood assays	30640-Apolipoprotein B (0.0)	-0.025	4.788E-05	4.320
blood assays	30650-Aspartate aminotransferase (0.0)	-0.029	2.337E-06	5.631
blood assays	30670-Urea (0.0)	-0.052	6.770E-18	17.169
blood assays	30680-Calcium (0.0)	-0.032	1.845E-07	6.734
blood assays	30690-Cholesterol (0.0)	-0.041	1.235E-11	10.908
blood assays	30720-Cystatin C (0.0)	-0.063	5.692E-25	24.245
blood assays	30730-Gamma glutamyltransferase (0.0)	-0.026	2.744E-05	4.562
blood assays	30740-Glucose (0.0)	-0.059	4.306E-22	21.366
blood assays	30750-Glycated haemoglobin (HbA1c) (0.0)	-0.066	2.790E-27	26.554
blood assays	30760-HDL cholesterol (0.0)	-0.047	8.444E-15	14.073
blood assays	30770-IGF-1 (0.0)	0.070	1.138E-30	29.944
blood assays	30780-LDL direct (0.0)	-0.026	1.396E-05	4.855

blood assays	30830-SHBG (0.0)	-0.030	1.200E-06	5.921
blood assays	30890-Vitamin D (0.0)	-0.039	1.009E-10	9.996
**DiaWAS**				
**cat_id**	**phe_name**	**r_value**	**p_value**	**-logp_value**
neoplasms	Skin cancer	-0.047	7.943E-15	14.100
neoplasms	Other non-epithelial cancer of skin	-0.046	2.169E-14	13.664
neoplasms	Cancer of prostate	-0.029	1.291E-06	5.889
neoplasms	Benign neoplasm of colon	-0.028	3.034E-06	5.518
endocrine/metabolic	Disorders of lipoid metabolism	-0.033	4.819E-08	7.317
endocrine/metabolic	Hyperlipidemia	-0.033	3.641E-08	7.439
endocrine/metabolic	Hypercholesterolemia	-0.032	2.058E-07	6.686
sense organs	Cataract	-0.055	2.501E-19	18.602
sense organs	Senile cataract	-0.043	1.433E-12	11.844
circulatory system	Hypertension	-0.077	3.832E-37	36.417
circulatory system	Essential hypertension	-0.077	3.677E-37	36.435
circulatory system	Ischemic Heart Disease	-0.026	2.364E-05	4.626
circulatory system	Cerebrovascular disease	-0.029	2.579E-06	5.589
digestive	Disorders of tooth development	0.029	1.678E-06	5.775
digestive	Disturbances in tooth eruption	0.029	2.327E-06	5.633
digestive	Diverticulosis and diverticulitis	-0.040	7.040E-11	10.152
digestive	Diverticulosis	-0.039	1.027E-10	9.988
genitourinary	Hyperplasia of prostate	-0.029	1.897E-06	5.722
genitourinary	Polyp of female genital organs	-0.027	1.156E-05	4.937
genitourinary	Polyp of corpus uteri	-0.026	1.577E-05	4.802
pregnancy complications	Miscarriage; stillbirth	0.043	8.556E-13	12.068
pregnancy complications	Missed abortion/Hydatidiform mole	0.027	6.192E-06	5.208
pregnancy complications	Hemorrhage during pregnancy; childbirth and postpartum	0.036	5.129E-09	8.290
pregnancy complications	Antepartum hemorrhage, abruptio placentae, and placenta previa	0.027	7.042E-06	5.152
pregnancy complications	Early or threatened labor; hemorrhage in early pregnancy	0.041	2.049E-11	10.688
pregnancy complications	Hemorrhage in early pregnancy	0.028	3.652E-06	5.437
pregnancy complications	Anemia during pregnancy	0.033	4.755E-08	7.323
pregnancy complications	Late pregnancy and failed induction	0.028	4.198E-06	5.377
pregnancy complications	Other complications of pregnancy NEC	0.040	3.123E-11	10.505
pregnancy complications	Normal delivery	0.041	1.131E-11	10.947
pregnancy complications	Malposition and malpresentation of fetus or obstruction	0.035	1.057E-08	7.976
pregnancy complications	Problems associated with amniotic cavity and membranes	0.041	1.723E-11	10.764
pregnancy complications	Abnormality of organs and soft tissues of pelvis complicating pregnancy, childbirth, or the puerperium	0.034	2.636E-08	7.579
pregnancy complications	Known or suspected fetal abnormality	0.051	6.338E-17	16.198

	affecting management of mother			
pregnancy complications	Fetal distress and abnormal forces of labor	0.047	5.603E-15	14.252
pregnancy complications	Umbilical cord complications during labor and delivery	0.028	4.024E-06	5.395
pregnancy complications	Obstetrical/birth trauma	0.062	2.019E-24	23.695
pregnancy complications	Complications of labor and delivery NEC	0.042	3.659E-12	11.437
**MedWAS**				
**cat_id**	**phe_name**	**r_value**	**p_value**	**-logp_value**
Alimentary Tract and Metabolism	A01A_Stomatological Preparations	-0.049	4.274E-16	15.369
Alimentary Tract and Metabolism	A06A_Drugs for Constipation	-0.023	1.144E-04	3.942
Blood and Blood Forming Organs	B01A_Antithrombotic Agents	-0.030	9.573E-07	6.019
Cardiovascular System	C01E_Other Cardiac Preparations	0.050	1.375E-16	15.862
Cardiovascular System	C03A_Low-Ceiling Diuretics, Thiazides	-0.056	5.662E-20	19.247
Cardiovascular System	C07A_Beta Blocking Agents	-0.043	2.271E-12	11.644
Cardiovascular System	C08C_Selective Calcium Channel Blockers with mainly Vascular Effects	-0.068	4.362E-29	28.360
Cardiovascular System	C09A_Ace Inhibitors, Plain	-0.055	1.831E-19	18.737
Cardiovascular System	C09C_Angiotensin Ii Receptor Blockers (Arbs), Plain	-0.041	1.024E-11	10.990
Cardiovascular System	C10A_Lipid Modifying Agents, Plain	-0.088	4.017E-47	46.396
Genito Urinary System and Sex Hormones	G02B_Contraceptives for Topical Use	0.025	5.242E-05	4.280
Genito Urinary System and Sex Hormones	G03A_Hormonal Contraceptives for Systemic Use	0.044	4.488E-13	12.348
Genito Urinary System and Sex Hormones	G04C_Drugs Used in Benign Prostatic Hypertrophy	-0.025	3.093E-05	4.510
Musculo-Skeletal System	M01A_Antiinflammatory and Antirheumatic Products, Non-Steroids	-0.028	5.403E-06	5.267
Musculo-Skeletal System	M05B_Drugs Affecting Bone Structure and Mineralization	-0.023	1.258E-04	3.900
Nervous System	N02B_Other Analgesics and Antipyretics	0.032	9.275E-08	7.033
Nervous System	N05C_Hypnotics and Sedatives	-0.023	1.267E-04	3.897
Sensory Organs	S01E_Antiglaucoma Preparations and Miotics	-0.026	1.333E-05	4.875
**Subtype 2**				
**PheWAS**				
**cat_id**	**phe_name**	**r_value**	**p_value**	**-logp_value**
lifestyle and environment - general	680_2.0-Own or rent accommodation lived in (0.0)	-0.026	2.002E-05	4.699
lifestyle and environment - general	6142#1-Current employment status (0.0)	-0.036	3.129E-09	8.505
lifestyle and environment - general	6142#2-Current employment status (0.0)	0.035	1.264E-08	7.898
lifestyle and environment - general	6141#2-How are people in household related to participant (0.0)	-0.036	2.054E-09	8.687

lifestyle and environment - general	6160#1-Leisure/social activities (0.0)	-0.044	7.314E-13	12.136
lifestyle and environment - general	709-Number in household (0.0)	-0.043	1.187E-12	11.925
lifestyle and environment - general	728-Number of vehicles in household (0.0)	-0.051	8.217E-17	16.085
lifestyle and environment - general	738-Average total household income before tax (0.0)	-0.052	1.620E-17	16.790
lifestyle and environment - general	2237-Plays computer games (0.0)	0.039	2.044E-10	9.689
lifestyle and environment - general	3-Verbal interview duration (0.0)	0.037	8.149E-10	9.089
lifestyle and environment - general	2139-Age first had sexual intercourse (0.0)	0.031	4.241E-07	6.372
lifestyle and environment - exercise and work	1150_2.0-Usual side of head for mobile phone use (0.0)	-0.026	1.430E-05	4.845
lifestyle and environment - exercise and work	6164#2-Types of physical activity in last 4 weeks (0.0)	-0.035	1.174E-08	7.930
lifestyle and environment - exercise and work	6164#3-Types of physical activity in last 4 weeks (0.0)	-0.039	1.213E-10	9.916
lifestyle and environment - exercise and work	864-Number of days/week walked 10+ minutes (0.0)	-0.032	1.334E-07	6.875
lifestyle and environment - exercise and work	904-Number of days/week of vigorous physical activity 10+ minutes (0.0)	-0.052	1.615E-17	16.792
lifestyle and environment - exercise and work	1110-Length of mobile phone use (0.0)	-0.034	3.176E-08	7.498
lifestyle and environment - exercise and work	1120-Weekly usage of mobile phone in last 3 months (0.0)	-0.046	6.166E-14	13.210
lifestyle and environment - exercise and work	1170-Getting up in morning (0.0)	-0.061	1.005E-23	22.998
lifestyle and environment - exercise and work	1180-Morning/evening person (chronotype) (0.0)	0.095	5.433E-55	54.265
lifestyle and environment - exercise and work	1220-Daytime dozing / sleeping (narcolepsy) (0.0)	-0.027	1.249E-05	4.904
lifestyle and environment - alcohol	1578-Average weekly champagne plus white wine intake (0.0)	-0.026	2.036E-05	4.691
lifestyle and environment - tobacco	3446_1.0-Type of tobacco currently smoked (0.0)	0.036	2.102E-09	8.677
lifestyle and environment - tobacco	20116_2.0-Smoking status (0.0)	0.037	1.745E-09	8.758
lifestyle and environment - tobacco	22506_3.0-Tobacco smoking (0.0)	0.026	1.671E-05	4.777
lifestyle and environment - tobacco	1239-Current tobacco smoking (0.0)	0.025	3.153E-05	4.501
lifestyle and environment - tobacco	20161-Pack years of smoking (0.0)	0.027	9.027E-06	5.044
physical measures - general	1707_1.0-Handedness (chirality/laterality) (0.0)	0.032	1.754E-07	6.756
physical measures - general	1707_2.0-Handedness (chirality/laterality) (0.0)	-0.036	4.466E-09	8.350
physical measures - cardiac & blood vessels	102-Pulse rate, automated reading (0.0)	0.046	5.441E-14	13.264
physical measures - cardiac & blood vessels	4079-Diastolic blood pressure, automated reading (0.0)	0.070	5.391E-31	30.268

physical measures - cardiac & blood vessels	4080-Systolic blood pressure, automated reading (0.0)	0.086	4.591E-45	44.338
physical measures - cardiac & blood vessels	12336-Ventricular rate (2.0)	0.065	2.383E-26	25.623
cognitive phenotypes	630-Touchscreen duration (0.0)	0.042	8.665E-12	11.062
cognitive phenotypes	6333-Duration spent answering each puzzle (2.0)	0.030	6.002E-07	6.222
cognitive phenotypes	20023-Mean time to correctly identify matches (0.0)	0.027	9.760E-06	5.011
cognitive phenotypes	20195-Number of symbol digit matches attempted (0.0)	-0.031	2.648E-07	6.577
cognitive phenotypes	23323-Number of symbol digit matches attempted (2.0)	-0.042	7.495E-12	11.125
cognitive phenotypes	23324-Number of symbol digit matches made correctly (2.0)	-0.032	1.561E-07	6.807
mental health self-report	2090-Seen doctor (GP) for nerves, anxiety, tension or depression (0.0)	0.038	3.431E-10	9.465
mental health self-report	20544#11-Mental health problems ever diagnosed by a professional (0.1)	0.027	1.238E-05	4.907
mental health self-report	20549#3-Substances taken for anxiety (0.1)	0.028	6.054E-06	5.218
mental health self-report	20458-General happiness (0.0)	0.031	3.570E-07	6.447
mental health self-report	20459-General happiness with own health (0.0)	0.042	4.777E-12	11.321
mental health self-report	20460-Belief that own life is meaningful (0.0)	-0.036	3.519E-09	8.454
mental health self-report	20489-Felt loved as a child (0.0)	-0.027	8.384E-06	5.077
blood assays	30710-C-reactive protein (0.0)	0.026	2.563E-05	4.591
blood assays	30740-Glucose (0.0)	0.029	1.726E-06	5.763
blood assays	30810-Phosphate (0.0)	-0.031	4.593E-07	6.338
blood assays	30890-Vitamin D (0.0)	-0.032	2.150E-07	6.667
		**DiaWAS**		
**cat_id**	**phe_name**	**r_value**	**p_value**	**-logp_value**
endocrine/metabolic	Diabetes mellitus	0.028	4.010E-06	5.397
endocrine/metabolic	Type 2 diabetes	0.028	2.904E-06	5.537
endocrine/metabolic	Protein-calorie malnutrition	0.029	2.629E-06	5.580
neurological	Multiple sclerosis	0.026	2.054E-05	4.687
circulatory system	Hypertension	0.074	8.091E-34	33.092
circulatory system	Essential hypertension	0.074	8.830E-34	33.054
circulatory system	Cardiac dysrhythmias	0.027	8.975E-06	5.047
circulatory system	Cerebrovascular disease	0.030	8.946E-07	6.048
circulatory system	Occlusion of cerebral arteries	0.027	8.849E-06	5.053
respiratory	Pneumonia due to fungus (mycoses)	0.027	8.002E-06	5.097
respiratory	Chronic airway obstruction	0.031	4.107E-07	6.386
respiratory	Chronic bronchitis	0.031	2.624E-07	6.581
respiratory	Obstructive chronic bronchitis	0.032	1.360E-07	6.866
		**MedWAS**		
**cat_id**	**phe_name**	**r_value**	**p_value**	**-logp_value**

Alimentary Tract and Metabolism	A01A_Stomatological Preparations	0.030	9.473E-07	6.024
Alimentary Tract and Metabolism	A02B_Drugs for Peptic Ulcer and Gastro-Oesophageal Reflux Disease (Gord)	0.030	1.224E-06	5.912
Alimentary Tract and Metabolism	A07E_Intestinal Antiinflammatory Agents	0.022	2.358E-04	3.627
Alimentary Tract and Metabolism	A10B_Blood Glucose Lowering Drugs, Excl. Insulins	0.024	7.035E-05	4.153
Blood and Blood Forming Organs	B01A_Antithrombotic Agents	0.032	2.039E-07	6.691
Cardiovascular System	C02C_ Antiadrenergic Agents, Peripherally Acting	0.022	2.940E-04	3.532
Cardiovascular System	C03A_Low-Ceiling Diuretics, Thiazides	0.034	1.829E-08	7.738
Cardiovascular System	C08C_Selective Calcium Channel Blockers with mainly Vascular Effects	0.062	1.337E-24	23.874
Cardiovascular System	C09A_Ace Inhibitors, Plain	0.056	3.100E-20	19.509
Cardiovascular System	C09C_Angiotensin Ii Receptor Blockers (Arbs), Plain	0.039	1.972E-10	9.705
Cardiovascular System	C10A_Lipid Modifying Agents, Plain	0.042	3.608E-12	11.443
Nervous System	N06A_Antidepressants	0.055	1.274E-19	18.895
**Subtype 3**				
**PheWAS**				
**cat_id**	**phe_name**	**r_value**	**p_value**	**-logp_value**
lifestyle and environment - general	20118_11.0-Home area population density - urban or rural (0.0)	-0.025	4.035E-05	4.394
lifestyle and environment - general	6138#4-Qualifications (0.0)	-0.044	4.016E-13	12.396
lifestyle and environment - general	6139#3-Gas or solid-fuel cooking/heating (0.0)	-0.026	1.753E-05	4.756
lifestyle and environment - general	6160#100-Leisure/social activities (0.0)	0.034	2.528E-08	7.597
lifestyle and environment - general	6160#1-Leisure/social activities (0.0)	-0.035	8.395E-09	8.076
lifestyle and environment - general	738-Average total household income before tax (0.0)	-0.038	6.681E-10	9.175
lifestyle and environment - general	2149-Lifetime number of sexual partners (0.0)	-0.038	3.184E-10	9.497
lifestyle and environment - general	3-Verbal interview duration (0.0)	-0.035	7.674E-09	8.115
lifestyle and environment - general	6-Conclusion duration (0.0)	-0.029	1.295E-06	5.888
lifestyle and environment - general	26414-Education score (England) (0.0)	0.045	2.237E-13	12.650
lifestyle and environment - exercise and work	6164#2-Types of physical activity in last 4 weeks (0.0)	-0.026	1.354E-05	4.868
lifestyle and environment - exercise and work	6164#3-Types of physical activity in last 4 weeks (0.0)	-0.030	6.793E-07	6.168
lifestyle and environment - exercise and work	816-Job involves heavy manual or physical work (0.0)	0.029	1.588E-06	5.799
lifestyle and environment - exercise and work	1070-Time spent watching television (TV) (0.0)	0.053	1.691E-18	17.772

lifestyle and environment - exercise and work	1100-Drive faster than motorway speed limit (0.0)	-0.042	3.475E-12	11.459
lifestyle and environment - exercise and work	1110-Length of mobile phone use (0.0)	-0.029	1.415E-06	5.849
lifestyle and environment - exercise and work	1120-Weekly usage of mobile phone in last 3 months (0.0)	-0.033	7.795E-08	7.108
lifestyle and environment - exercise and work	1170-Getting up in morning (0.0)	0.031	5.260E-07	6.279
lifestyle and environment - exercise and work	1180-Morning/evening person (chronotype) (0.0)	-0.047	1.191E-14	13.924
lifestyle and environment - alcohol	20405_0.0-Ever had known person concerned about, or recommend reduction of, alcohol consumption (0.0)	0.029	1.273E-06	5.895
lifestyle and environment - alcohol	1558-Alcohol intake frequency. (0.0)	-0.031	2.946E-07	6.531
lifestyle and environment - alcohol	1568-Average weekly red wine intake (0.0)	-0.028	2.796E-06	5.554
lifestyle and environment - alcohol	20414-Frequency of drinking alcohol (0.0)	-0.034	2.507E-08	7.601
lifestyle and environment - tobacco	22506_0.0-Tobacco smoking (0.0)	0.028	3.570E-06	5.447
physical measures - general	23099-Body fat percentage (0.0)	0.026	1.796E-05	4.746
physical measures - general	23106-Impedance of whole body (0.0)	0.034	3.476E-08	7.459
physical measures - general	23107-Impedance of leg (right) (0.0)	0.033	4.953E-08	7.305
physical measures - general	23108-Impedance of leg (left) (0.0)	0.036	5.081E-09	8.294
physical measures - cardiac & blood vessels	102-Pulse rate, automated reading (0.0)	0.030	6.329E-07	6.199
physical measures - cardiac & blood vessels	12336-Ventricular rate (2.0)	0.050	2.359E-16	15.627
physical measures - cardiac & blood vessels	12338-P duration (2.0)	-0.026	1.702E-05	4.769
physical measures - cardiac & blood vessels	22670-Minimum carotid IMT (intima-medial thickness) at 120 degrees (2.0)	-0.042	2.897E-12	11.538
physical measures - cardiac & blood vessels	22671-Mean carotid IMT (intima-medial thickness) at 120 degrees (2.0)	-0.046	2.040E-14	13.690
physical measures - cardiac & blood vessels	22672-Maximum carotid IMT (intima-medial thickness) at 120 degrees (2.0)	-0.040	6.627E-11	10.179
physical measures - cardiac & blood vessels	22673-Minimum carotid IMT (intima-medial thickness) at 150 degrees (2.0)	-0.041	1.013E-11	10.995
physical measures - cardiac & blood vessels	22674-Mean carotid IMT (intima-medial thickness) at 150 degrees (2.0)	-0.041	1.157E-11	10.937
physical measures - cardiac & blood vessels	22675-Maximum carotid IMT (intima-medial thickness) at 150 degrees (2.0)	-0.037	1.679E-09	8.775
physical measures - cardiac & blood vessels	22676-Minimum carotid IMT (intima-medial thickness) at 210 degrees (2.0)	-0.032	1.687E-07	6.773

physical measures - cardiac & blood vessels	22677-Mean carotid IMT (intima-medial thickness) at 210 degrees (2.0)	-0.035	6.167E-09	8.210
physical measures - cardiac & blood vessels	22678-Maximum carotid IMT (intima-medial thickness) at 210 degrees (2.0)	-0.031	3.814E-07	6.419
physical measures - cardiac & blood vessels	22679-Minimum carotid IMT (intima-medial thickness) at 240 degrees (2.0)	-0.033	6.964E-08	7.157
physical measures - cardiac & blood vessels	22680-Mean carotid IMT (intima-medial thickness) at 240 degrees (2.0)	-0.034	2.895E-08	7.538
physical measures - cardiac & blood vessels	22681-Maximum carotid IMT (intima-medial thickness) at 240 degrees (2.0)	-0.032	1.406E-07	6.852
cognitive phenotypes	6374-Number of puzzles viewed (2.0)	0.025	2.958E-05	4.529
cognitive phenotypes	6383-Number of puzzles attempted (2.0)	0.028	3.135E-06	5.504
cognitive phenotypes	400-Time to complete round (0.1)	-0.025	2.994E-05	4.524
cognitive phenotypes	6348-Duration to complete numeric path (trail #1) (2.0)	-0.029	1.540E-06	5.813
cognitive phenotypes	6350-Duration to complete alphanumeric path (trail #2) (2.0)	-0.026	2.425E-05	4.615
cognitive phenotypes	6772-Interval between previous point and current one in numeric path (trail #1) (2.0)	-0.031	2.769E-07	6.558
mental health self-report	20529_0.0-Victim of physically violent crime (0.0)	0.025	3.468E-05	4.460
mental health self-report	1970-Nervous feelings (0.0)	0.034	2.250E-08	7.648
mental health self-report	1980-Worrier / anxious feelings (0.0)	0.027	9.229E-06	5.035
mental health self-report	2040-Risk taking (0.0)	-0.054	6.609E-19	18.180
**DiaWAS**				
**cat_id**	**phe_name**	**r_value**	**p_value**	**-logp_value**
circulatory system	Hypertension	-0.034	3.328E-08	7.478
circulatory system	Essential hypertension	-0.034	2.784E-08	7.555
**MedWAS**				
**cat_id**	**phe_name**	**r_value**	**p_value**	**-logp_value**
Alimentary Tract and Metabolism	A01A_Stomatological Preparations	-0.035	6.806E-09	8.167
Cardiovascular System	C09A_Ace Inhibitors, Plain	-0.022	3.329E-04	3.478
**Subtype 4**				
**PheWAS**				
**cat_id**	**phe_name**	**r_value**	**p_value**	**-logp_value**
lifestyle and environment - general	2395_1.0-Hair/balding pattern (0.0)	-0.039	2.385E-10	9.622
lifestyle and environment - general	2395_2.0-Hair/balding pattern (0.0)	-0.031	2.698E-07	6.569
lifestyle and environment - general	2395_3.0-Hair/balding pattern (0.0)	-0.044	2.942E-13	12.531
lifestyle and environment - general	2395_4.0-Hair/balding pattern (0.0)	-0.044	4.671E-13	12.331
lifestyle and environment - general	6142#1-Current employment status (0.0)	0.033	3.714E-08	7.430
lifestyle and environment - general	6142#2-Current employment status (0.0)	-0.042	4.884E-12	11.311

lifestyle and environment - general	6160#2-Leisure/social activities (0.0)	-0.035	1.026E-08	7.989
lifestyle and environment - general	6160#5-Leisure/social activities (0.0)	0.026	1.810E-05	4.742
lifestyle and environment - general	3-Verbal interview duration (0.0)	0.025	3.534E-05	4.452
lifestyle and environment - general	767-Length of working week for main job (0.0)	-0.026	2.242E-05	4.649
lifestyle and environment - exercise and work	943-Frequency of stair climbing in last 4 weeks (0.0)	0.025	2.780E-05	4.556
lifestyle and environment - exercise and work	1070-Time spent watching television (TV) (0.0)	-0.032	1.281E-07	6.892
lifestyle and environment - exercise and work	1180-Morning/evening person (chronotype) (0.0)	-0.035	1.124E-08	7.949
lifestyle and environment - alcohol	1558-Alcohol intake frequency. (0.0)	-0.028	5.981E-06	5.223
lifestyle and environment - alcohol	1588-Average weekly beer plus cider intake (0.0)	-0.048	2.695E-15	14.569
lifestyle and environment - alcohol	20403-Amount of alcohol drunk on a typical drinking day (0.0)	-0.025	3.958E-05	4.403
lifestyle and environment - alcohol	20416-Frequency of consuming six or more units of alcohol (0.0)	-0.034	2.939E-08	7.532
physical measures - general	1707_1.0-Handedness (chirality/laterality) (0.0)	0.036	2.230E-09	8.652
physical measures - general	1707_2.0-Handedness (chirality/laterality) (0.0)	-0.035	1.248E-08	7.904
physical measures - general	46-Hand grip strength (left) (0.0)	-0.062	4.057E-24	23.392
physical measures - general	47-Hand grip strength (right) (0.0)	-0.051	3.122E-17	16.506
physical measures - general	48-Waist circumference (0.0)	-0.043	2.233E-12	11.651
physical measures - general	50-Standing height (0.0)	-0.060	4.391E-23	22.357
physical measures - general	3062-Forced vital capacity (FVC) (0.0)	-0.048	1.741E-15	14.759
physical measures - general	3063-Forced expiratory volume in 1-second (FEV1) (0.0)	-0.040	4.142E-11	10.383
physical measures - general	3064-Peak expiratory flow (PEF) (0.0)	-0.027	1.204E-05	4.920
physical measures - general	12143-Weight (pre-imaging) (2.0)	-0.038	3.637E-10	9.439
physical measures - general	12144-Height (2.0)	-0.062	2.366E-24	23.626
physical measures - general	20015-Sitting height (0.0)	-0.050	1.844E-16	15.734
physical measures - general	21002-Weight (0.0)	-0.041	1.461E-11	10.835
physical measures - general	23099-Body fat percentage (0.0)	0.059	3.012E-22	21.521
physical measures - general	23100-Whole body fat mass (0.0)	0.026	2.383E-05	4.623
physical measures - general	23101-Whole body fat-free mass (0.0)	-0.062	2.428E-24	23.615
physical measures - general	23102-Whole body water mass (2.0)	-0.062	9.224E-25	24.035
physical measures - general	23106-Impedance of whole body (0.0)	0.050	1.802E-16	15.744
physical measures - general	23109-Impedance of arm (right) (0.0)	0.061	1.941E-23	22.712
physical measures - general	23110-Impedance of arm (left) (0.0)	0.062	1.533E-24	23.814

physical measures - general	23111-Leg fat percentage (right) (0.0)	0.073	2.066E-33	32.685
physical measures - general	23112-Leg fat mass (right) (0.0)	0.067	2.043E-28	27.690
physical measures - general	23113-Leg fat-free mass (right) (0.0)	-0.058	1.969E-21	20.706
physical measures - general	23119-Arm fat percentage (right) (0.0)	0.060	3.713E-23	22.430
physical measures - general	23121-Arm fat-free mass (right) (0.0)	-0.063	1.573E-25	24.803
physical measures - general	23123-Arm fat percentage (left) (0.0)	0.062	2.446E-24	23.612
physical measures - general	23125-Arm fat-free mass (left) (0.0)	-0.065	5.129E-27	26.290
physical measures - general	23126-Arm predicted mass (left) (0.0)	-0.065	1.233E-26	25.909
physical measures - general	23127-Trunk fat percentage (0.0)	0.035	1.335E-08	7.875
physical measures - cardiac & blood vessels	102-Pulse rate, automated reading (0.0)	0.030	8.163E-07	6.088
cognitive phenotypes	6373-Number of puzzles correctly solved (2.0)	0.033	3.949E-08	7.404
cognitive phenotypes	400-Time to complete round (0.1)	-0.025	4.929E-05	4.307
cognitive phenotypes	404-Duration to first press of snap-button in each round (0.0)	-0.044	7.791E-13	12.108
cognitive phenotypes	6772-Interval between previous point and current one in numeric path (trail #1) (2.0)	-0.025	3.074E-05	4.512
cognitive phenotypes	6773-Interval between previous point and current one in alphanumeric path (trail #2) (2.0)	-0.026	2.475E-05	4.606
cognitive phenotypes	20023-Mean time to correctly identify matches (0.0)	-0.037	1.036E-09	8.985
cognitive phenotypes	20159-Number of symbol digit matches made correctly (0.0)	0.025	4.894E-05	4.310
cognitive phenotypes	23324-Number of symbol digit matches made correctly (2.0)	0.027	7.656E-06	5.116
mental health self-report	20531_1.0-Victim of sexual assault (0.0)	0.030	1.154E-06	5.938
mental health self-report	1930-Miserableness (0.0)	0.033	7.360E-08	7.133
mental health self-report	2090-Seen doctor (GP) for nerves, anxiety, tension or depression (0.0)	0.031	2.391E-07	6.622
mental health self-report	20441-Ever had prolonged loss of interest in normal activities (0.0)	0.029	1.357E-06	5.867
mental health self-report	20446-Ever had prolonged feelings of sadness or depression (0.0)	0.028	4.889E-06	5.311
mental health self-report	20547#1-Activities undertaken to treat depression (0.1)	0.025	3.587E-05	4.445
mental health self-report	2080-Frequency of tiredness / lethargy in last 2 weeks (0.0)	0.035	8.224E-09	8.085
physical measures - bone density and sizes	4095-Heel ultrasound method (right) (2.0)	0.027	6.999E-06	5.155

physical measures - bone density and sizes	78-Heel bone mineral density (BMD) T-score, automated (0.0)	-0.027	9.100E-06	5.041
physical measures - bone density and sizes	3143-Ankle spacing width (0.0)	-0.025	5.110E-05	4.292
physical measures - bone density and sizes	3144-Heel Broadband ultrasound attenuation, direct entry (0.0)	-0.040	4.925E-11	10.308
physical measures - bone density and sizes	4101-Heel broadband ultrasound attenuation (left) (0.0)	-0.025	4.452E-05	4.351
blood assays	30010-Red blood cell (erythrocyte) count (0.0)	-0.051	7.809E-17	16.107
blood assays	30020-Haemoglobin concentration (0.0)	-0.070	4.183E-31	30.379
blood assays	30030-Haematocrit percentage (0.0)	-0.066	2.241E-27	26.650
blood assays	30050-Mean corpuscular haemoglobin (0.0)	-0.030	1.009E-06	5.996
blood assays	30080-Platelet count (0.0)	0.036	4.569E-09	8.340
blood assays	30090-Platelet crit (0.0)	0.037	1.362E-09	8.866
blood assays	30130-Monocyte count (0.0)	-0.027	1.033E-05	4.986
blood assays	30630-Apolipoprotein A (0.0)	0.025	4.433E-05	4.353
blood assays	30650-Aspartate aminotransferase (0.0)	-0.027	1.313E-05	4.882
blood assays	30660-Direct bilirubin (0.0)	-0.036	1.993E-09	8.701
blood assays	30700-Creatinine (0.0)	-0.076	5.844E-36	35.233
blood assays	30720-Cystatin C (0.0)	-0.045	1.297E-13	12.887
blood assays	30730-Gamma glutamyltransferase (0.0)	-0.043	9.610E-13	12.017
blood assays	30760-HDL cholesterol (0.0)	0.030	1.038E-06	5.984
blood assays	30810-Phosphate (0.0)	0.026	1.441E-05	4.841
blood assays	30830-SHBG (0.0)	0.037	1.541E-09	8.812
blood assays	30840-Total bilirubin (0.0)	-0.037	7.173E-10	9.144
blood assays	30850-Testosterone (0.0)	-0.083	9.387E-43	42.027
blood assays	30880-Urate (0.0)	-0.078	1.793E-37	36.747
**DiaWAS**				
**cat_id**	**phe_name**	**r_value**	**p_value**	**-logp_value**
digestive	Inguinal hernia	-0.031	3.763E-07	6.424
genitourinary	Disorders of menstruation and other abnormal bleeding from female genital tract	0.026	1.377E-05	4.861
musculoskeletal	Disorders of muscle, ligament, and fascia	-0.028	4.363E-06	5.360
musculoskeletal	Fasciitis	-0.026	2.429E-05	4.615
musculoskeletal	Contracture of palmar fascia [Dupuytren's disease]	-0.028	3.633E-06	5.440
**MedWAS**				
**cat_id**	**phe_name**	**r_value**	**p_value**	**-logp_value**
Alimentary Tract and Metabolism	A12A_Calcium	0.025	3.418E-05	4.466
Nervous System	N02B_Other Analgesics and Antipyretics	0.024	6.649E-05	4.177
Nervous System	N06A_Antidepressants	0.032	2.042E-07	6.690
**Subtype 5**				
**PheWAS**				
**cat_id**	**phe_name**	**r_value**	**p_value**	**-logp_value**
lifestyle and environment - general	680_2.0-Own or rent accommodation lived in (0.0)	-0.025	4.958E-05	4.305

lifestyle and environment - general	2395_1.0-Hair/balding pattern (0.0)	0.062	4.232E-24	23.373
lifestyle and environment - general	2395_2.0-Hair/balding pattern (0.0)	0.070	1.964E-30	29.707
lifestyle and environment - general	2395_3.0-Hair/balding pattern (0.0)	0.089	7.428E-49	48.129
lifestyle and environment - general	2395_4.0-Hair/balding pattern (0.0)	0.066	3.947E-27	26.404
lifestyle and environment - general	1797-Father still alive (0.0)	-0.047	1.180E-14	13.928
lifestyle and environment - general	1835-Mother still alive (0.0)	-0.047	5.683E-15	14.245
lifestyle and environment - general	6138#4-Qualifications (0.0)	0.026	1.787E-05	4.748
lifestyle and environment - general	6138#2-Qualifications (0.0)	-0.027	1.165E-05	4.934
lifestyle and environment - general	6142#2-Current employment status (0.0)	0.040	5.291E-11	10.276
lifestyle and environment - general	6142#3-Current employment status (0.0)	-0.048	3.492E-15	14.457
lifestyle and environment - general	6141#2-How are people in household related to participant (0.0)	-0.037	9.598E-10	9.018
lifestyle and environment - general	6145#2-Illness, injury, bereavement, stress in last 2 years (0.0)	-0.026	1.954E-05	4.709
lifestyle and environment - general	6160#2-Leisure/social activities (0.0)	0.038	3.049E-10	9.516
lifestyle and environment - general	709-Number in household (0.0)	-0.035	7.612E-09	8.118
lifestyle and environment - general	1031-Frequency of friend/family visits (0.0)	0.032	1.597E-07	6.797
lifestyle and environment - general	4-Biometrics duration (0.0)	0.028	4.205E-06	5.376
lifestyle and environment - general	767-Length of working week for main job (0.0)	0.036	2.715E-09	8.566
lifestyle and environment - general	2139-Age first had sexual intercourse (0.0)	0.036	2.601E-09	8.585
lifestyle and environment - general	24003-Nitrogen dioxide air pollution; 2010 (0.0)	0.026	1.544E-05	4.811
lifestyle and environment - general	24016-Nitrogen dioxide air pollution; 2005 (0.0)	0.027	1.282E-05	4.892
lifestyle and environment - general	24017-Nitrogen dioxide air pollution; 2006 (0.0)	0.026	1.549E-05	4.810
lifestyle and environment - general	24018-Nitrogen dioxide air pollution; 2007 (0.0)	0.025	3.975E-05	4.401
lifestyle and environment - exercise and work	1210-Snoring (0.0)	0.025	4.660E-05	4.332
lifestyle and environment - exercise and work	6164#3-Types of physical activity in last 4 weeks (0.0)	0.036	4.549E-09	8.342
lifestyle and environment - exercise and work	904-Number of days/week of vigorous physical activity 10+ minutes (0.0)	0.041	2.167E-11	10.664
lifestyle and environment - exercise and work	1100-Drive faster than motorway speed limit (0.0)	0.036	2.921E-09	8.534
lifestyle and environment - exercise and work	1170-Getting up in morning (0.0)	0.027	1.069E-05	4.971
lifestyle and environment - alcohol	20405_0.0-Ever had known person concerned about, or recommend reduction of, alcohol consumption (0.0)	-0.025	3.819E-05	4.418
lifestyle and environment - alcohol	1558-Alcohol intake frequency. (0.0)	0.037	9.044E-10	9.044

lifestyle and environment - alcohol	1588-Average weekly beer plus cider intake (0.0)	0.073	3.911E-33	32.408
lifestyle and environment - tobacco	2877_3.0-Type of tobacco previously smoked (0.0)	0.031	4.620E-07	6.335
lifestyle and environment - tobacco	20116_0.0-Smoking status (0.0)	-0.028	3.809E-06	5.419
lifestyle and environment - tobacco	20116_1.0-Smoking status (0.0)	0.025	4.049E-05	4.393
lifestyle and environment - tobacco	20160-Ever smoked (0.0)	0.031	3.717E-07	6.430
lifestyle and environment - tobacco	1249-Past tobacco smoking (0.0)	-0.031	3.665E-07	6.436
physical measures - general	1707_1.0-Handedness (chirality/laterality) (0.0)	-0.045	2.314E-13	12.636
physical measures - general	1707_2.0-Handedness (chirality/laterality) (0.0)	0.043	1.061E-12	11.974
physical measures - general	46-Hand grip strength (left) (0.0)	0.113	4.347E-77	76.362
physical measures - general	47-Hand grip strength (right) (0.0)	0.109	7.376E-72	71.132
physical measures - general	48-Waist circumference (0.0)	0.078	2.151E-37	36.667
physical measures - general	50-Standing height (0.0)	0.117	2.135E-82	81.671
physical measures - general	3062-Forced vital capacity (FVC) (0.0)	0.100	3.608E-61	60.443
physical measures - general	3063-Forced expiratory volume in 1-second (FEV1) (0.0)	0.087	8.500E-47	46.071
physical measures - general	3064-Peak expiratory flow (PEF) (0.0)	0.082	3.155E-41	40.501
physical measures - general	12143-Weight (pre-imaging) (2.0)	0.059	1.605E-22	21.795
physical measures - general	12144-Height (2.0)	0.115	9.453E-80	79.024
physical measures - general	20015-Sitting height (0.0)	0.105	5.329E-67	66.273
physical measures - general	21002-Weight (0.0)	0.071	2.449E-31	30.611
physical measures - general	23099-Body fat percentage (0.0)	-0.130	1.134E-101	100.946
physical measures - general	23100-Whole body fat mass (0.0)	-0.066	2.068E-27	26.684
physical measures - general	23101-Whole body fat-free mass (0.0)	0.126	1.543E-95	94.812
physical measures - general	23102-Whole body water mass (2.0)	0.119	1.985E-86	85.702
physical measures - general	23106-Impedance of whole body (0.0)	-0.119	3.514E-85	84.454
physical measures - general	23107-Impedance of leg (right) (0.0)	-0.081	7.045E-41	40.152
physical measures - general	23108-Impedance of leg (left) (0.0)	-0.075	5.261E-35	34.279
physical measures - general	23109-Impedance of arm (right) (0.0)	-0.125	1.894E-94	93.723
physical measures - general	23110-Impedance of arm (left) (0.0)	-0.128	2.001E-99	98.699
physical measures - general	23111-Leg fat percentage (right) (0.0)	-0.149	1.684E-133	132.774
physical measures - general	23112-Leg fat mass (right) (0.0)	-0.134	4.237E-109	108.373
physical measures - general	23113-Leg fat-free mass (right) (0.0)	0.121	3.715E-88	87.430
physical measures - general	23119-Arm fat percentage (right) (0.0)	-0.131	4.368E-104	103.360
physical measures - general	23120-Arm fat mass (right) (0.0)	-0.061	1.159E-23	22.936

physical measures - general	23121-Arm fat-free mass (right) (0.0)	0.125	3.881E-95	94.411
physical measures - general	23123-Arm fat percentage (left) (0.0)	-0.138	3.071E-114	113.513
physical measures - general	23125-Arm fat-free mass (left) (0.0)	0.126	1.073E-96	95.969
physical measures - general	23126-Arm predicted mass (left) (0.0)	0.128	4.264E-99	98.370
physical measures - general	23127-Trunk fat percentage (0.0)	-0.086	5.469E-46	45.262
physical measures - cardiac & blood vessels	102-Pulse rate, automated reading (0.0)	-0.031	3.828E-07	6.417
physical measures - cardiac & blood vessels	4079-Diastolic blood pressure, automated reading (0.0)	0.048	2.648E-15	14.577
physical measures - cardiac & blood vessels	4080-Systolic blood pressure, automated reading (0.0)	0.066	4.008E-27	26.397
physical measures - cardiac & blood vessels	4195-Pulse wave reflection index (0.0)	0.026	2.382E-05	4.623
physical measures - cardiac & blood vessels	12340-QRS duration (2.0)	0.055	1.306E-19	18.884
physical measures - cardiac & blood vessels	21021-Pulse wave Arterial Stiffness index (0.0)	0.026	2.040E-05	4.690
physical measures - cardiac & blood vessels	22670-Minimum carotid IMT (intima-medial thickness) at 120 degrees (2.0)	0.042	4.187E-12	11.378
physical measures - cardiac & blood vessels	22671-Mean carotid IMT (intima-medial thickness) at 120 degrees (2.0)	0.050	1.335E-16	15.874
physical measures - cardiac & blood vessels	22672-Maximum carotid IMT (intima-medial thickness) at 120 degrees (2.0)	0.047	1.935E-14	13.713
physical measures - cardiac & blood vessels	22673-Minimum carotid IMT (intima-medial thickness) at 150 degrees (2.0)	0.044	8.126E-13	12.090
physical measures - cardiac & blood vessels	22674-Mean carotid IMT (intima-medial thickness) at 150 degrees (2.0)	0.040	5.595E-11	10.252
physical measures - cardiac & blood vessels	22675-Maximum carotid IMT (intima-medial thickness) at 150 degrees (2.0)	0.035	8.386E-09	8.076
physical measures - cardiac & blood vessels	22676-Minimum carotid IMT (intima-medial thickness) at 210 degrees (2.0)	0.047	1.707E-14	13.768
physical measures - cardiac & blood vessels	22677-Mean carotid IMT (intima-medial thickness) at 210 degrees (2.0)	0.051	4.114E-17	16.386
physical measures - cardiac & blood vessels	22678-Maximum carotid IMT (intima-medial thickness) at 210 degrees (2.0)	0.046	6.560E-14	13.183
physical measures - cardiac & blood vessels	22679-Minimum carotid IMT (intima-medial thickness) at 240 degrees (2.0)	0.052	5.944E-18	17.226
physical measures - cardiac & blood vessels	22680-Mean carotid IMT (intima-medial thickness) at 240 degrees (2.0)	0.060	2.451E-23	22.611
physical measures - cardiac & blood vessels	22681-Maximum carotid IMT (intima-medial	0.057	4.925E-21	20.308

	thickness) at 240 degrees (2.0)			
cognitive phenotypes	20152-Reproduciblity of spirometry measurement using ERS/ATS criteria (0.0)	-0.030	1.161E-06	5.935
cognitive phenotypes	399-Number of incorrect matches in round (0.1)	0.030	7.580E-07	6.120
cognitive phenotypes	400-Time to complete round (0.1)	0.034	1.452E-08	7.838
cognitive phenotypes	4290-Duration screen displayed (0.0)	0.027	8.841E-06	5.054
cognitive phenotypes	6348-Duration to complete numeric path (trail #1) (2.0)	0.047	1.360E-14	13.867
cognitive phenotypes	6350-Duration to complete alphanumeric path (trail #2) (2.0)	0.035	1.398E-08	7.854
cognitive phenotypes	6772-Interval between previous point and current one in numeric path (trail #1) (2.0)	0.042	5.265E-12	11.279
cognitive phenotypes	6773-Interval between previous point and current one in alphanumeric path (trail #2) (2.0)	0.035	8.889E-09	8.051
cognitive phenotypes	20133-Time to complete round (0.0)	0.033	5.998E-08	7.222
cognitive phenotypes	20153-Forced expiratory volume in 1-second (FEV1), predicted (0.0)	0.038	2.484E-10	9.605
cognitive phenotypes	20156-Duration to complete numeric path (trail #1) (0.0)	0.029	2.317E-06	5.635
cognitive phenotypes	20159-Number of symbol digit matches made correctly (0.0)	-0.029	1.780E-06	5.750
cognitive phenotypes	20195-Number of symbol digit matches attempted (0.0)	-0.026	2.340E-05	4.631
cognitive phenotypes	23323-Number of symbol digit matches attempted (2.0)	-0.033	5.784E-08	7.238
cognitive phenotypes	23324-Number of symbol digit matches made correctly (2.0)	-0.026	2.547E-05	4.594
mental health self-report	20531_1.0-Victim of sexual assault (0.0)	-0.026	1.414E-05	4.850
mental health self-report	20536_2.0-Weight change during worst episode of depression (0.0)	-0.037	1.855E-09	8.732
mental health self-report	1930-Miserableness (0.0)	-0.039	1.821E-10	9.740
mental health self-report	1950-Sensitivity / hurt feelings (0.0)	-0.034	2.139E-08	7.670
mental health self-report	1960-Fed-up feelings (0.0)	-0.029	2.210E-06	5.656
mental health self-report	1980-Worrier / anxious feelings (0.0)	-0.028	3.676E-06	5.435
mental health self-report	2000-Worry too long after embarrassment (0.0)	-0.034	2.155E-08	7.667
mental health self-report	2030-Guilty feelings (0.0)	-0.040	5.932E-11	10.227
mental health self-report	2040-Risk taking (0.0)	0.038	3.197E-10	9.495
mental health self-report	2090-Seen doctor (GP) for nerves, anxiety,	-0.030	7.164E-07	6.145

	tension or depression (0.0)			
mental health self-report	20446-Ever had prolonged feelings of sadness or depression (0.0)	-0.025	3.532E-05	4.452
mental health self-report	2080-Frequency of tiredness / lethargy in last 2 weeks (0.0)	-0.032	1.618E-07	6.791
mental health self-report	2110-Able to confide (0.0)	-0.025	3.609E-05	4.443
mental health self-report	20453-Ever taken cannabis (0.0)	0.025	3.422E-05	4.466
mental health self-report	20511-Recent poor appetite or overeating (0.0)	-0.028	5.450E-06	5.264
mental health self-report	20520-Recent worrying too much about different things (0.0)	-0.026	1.587E-05	4.799
mental health self-report	20521-Belittlement by partner or ex-partner as an adult (0.0)	-0.026	1.511E-05	4.821
physical measures - bone density and sizes	3143-Ankle spacing width (0.0)	0.048	2.224E-15	14.653
physical measures - bone density and sizes	3144-Heel Broadband ultrasound attenuation, direct entry (0.0)	0.031	4.506E-07	6.346
physical measures - bone density and sizes	4100-Ankle spacing width (left) (0.0)	0.041	1.458E-11	10.836
physical measures - bone density and sizes	4119-Ankle spacing width (right) (0.0)	0.032	1.845E-07	6.734
blood assays	30150-Eosinophill count (0.0)	0.026	1.418E-05	4.848
blood assays	30010-Red blood cell (erythrocyte) count (0.0)	0.091	2.058E-50	49.687
blood assays	30020-Haemoglobin concentration (0.0)	0.113	6.008E-78	77.221
blood assays	30030-Haematocrit percentage (0.0)	0.116	4.330E-82	81.364
blood assays	30040-Mean corpuscular volume (0.0)	0.040	3.100E-11	10.509
blood assays	30050-Mean corpuscular haemoglobin (0.0)	0.036	3.008E-09	8.522
blood assays	30080-Platelet count (0.0)	-0.045	1.322E-13	12.879
blood assays	30090-Platelet crit (0.0)	-0.061	7.608E-24	23.119
blood assays	30130-Monocyte count (0.0)	0.033	6.135E-08	7.212
blood assays	30190-Monocyte percentage (0.0)	0.043	1.060E-12	11.975
blood assays	30620-Alanine aminotransferase (0.0)	0.072	2.070E-32	31.684
blood assays	30630-Apolipoprotein A (0.0)	-0.030	5.732E-07	6.242
blood assays	30650-Aspartate aminotransferase (0.0)	0.073	3.601E-33	32.444
blood assays	30660-Direct bilirubin (0.0)	0.039	2.017E-10	9.695
blood assays	30670-Urea (0.0)	0.046	2.686E-14	13.571
blood assays	30700-Creatinine (0.0)	0.106	2.711E-68	67.567
blood assays	30720-Cystatin C (0.0)	0.055	2.300E-19	18.638
blood assays	30730-Gamma glutamyltransferase (0.0)	0.062	9.338E-25	24.030
blood assays	30760-HDL cholesterol (0.0)	-0.040	5.693E-11	10.245
blood assays	30810-Phosphate (0.0)	-0.035	1.005E-08	7.998
blood assays	30830-SHBG (0.0)	-0.048	1.928E-15	14.715
blood assays	30840-Total bilirubin (0.0)	0.051	2.554E-17	16.593

blood assays	30850-Testosterone (0.0)	0.133	1.340E-106	105.873
blood assays	30870-Triglycerides (0.0)	0.039	9.827E-11	10.008
blood assays	30880-Urate (0.0)	0.095	1.114E-54	53.953
**DiaWAS**				
**cat_id**	**phe_name**	**r_value**	**p_value**	**-logp_value**
neoplasms	Breast cancer	-0.026	2.718E-05	4.566
neoplasms	Cancer of prostate	0.033	6.510E-08	7.186
neoplasms	Benign neoplasm of uterus	-0.034	1.837E-08	7.736
neoplasms	Uterine leiomyoma	-0.034	3.023E-08	7.519
circulatory system	Hypertension	0.033	8.381E-08	7.077
circulatory system	Essential hypertension	0.032	9.440E-08	7.025
circulatory system	Atrial fibrillation and flutter	0.027	6.345E-06	5.198
digestive	Inguinal hernia	0.041	1.807E-11	10.743
genitourinary	Hyperplasia of prostate	0.044	5.027E-13	12.299
genitourinary	Genital prolapse	-0.035	1.022E-08	7.991
genitourinary	Prolapse of vaginal walls	-0.035	1.323E-08	7.879
genitourinary	Noninflammatory female genital disorders	-0.041	9.257E-12	11.034
genitourinary	Noninflammatory disorders of ovary, fallopian tube, and broad ligament	-0.026	1.584E-05	4.800
genitourinary	Disorders of uterus, NEC	-0.026	1.815E-05	4.741
genitourinary	stress incontinence, female	-0.028	5.411E-06	5.267
genitourinary	Disorders of menstruation and other abnormal bleeding from female genital tract	-0.048	2.766E-15	14.558
genitourinary	Irregular menstrual cycle/bleeding	-0.046	6.843E-14	13.165
genitourinary	Excessive or frequent menstruation	-0.040	2.729E-11	10.564
genitourinary	Menopausal and postmenopausal disorders	-0.025	3.063E-05	4.514
genitourinary	Ovarian cyst	-0.026	2.047E-05	4.689
pregnancy complications	Early or threatened labor; hemorrhage in early pregnancy	-0.027	1.036E-05	4.985
pregnancy complications	Normal delivery	-0.035	1.357E-08	7.867
pregnancy complications	Known or suspected fetal abnormality affecting management of mother	-0.034	2.547E-08	7.594
pregnancy complications	Obstetrical/birth trauma	-0.043	8.939E-13	12.049
pregnancy complications	Complications of labor and delivery NEC	-0.027	9.185E-06	5.037
symptoms	Abdominal pain	-0.025	2.848E-05	4.545
**MedWAS**				
**cat_id**	**phe_name**	**r_value**	**p_value**	**-logp_value**
Alimentary Tract and Metabolism	A01A_Stomatological Preparations	0.032	1.763E-07	6.754
Blood and Blood Forming Organs	B01A_Antithrombotic Agents	0.029	2.769E-06	5.558
Cardiovascular System	C01A_Cardiac Glycosides	0.030	1.204E-06	5.920
Cardiovascular System	C01E_Other Cardiac Preparations	-0.037	1.810E-09	8.742
Cardiovascular System	C07A_Beta Blocking Agents	0.023	1.531E-04	3.815

Cardiovascular System	C08C_Selective Calcium Channel Blockers with mainly Vascular Effects	0.034	2.974E-08	7.527
Cardiovascular System	C09A_Ace Inhibitors, Plain	0.036	4.095E-09	8.388
Cardiovascular System	C09C_Angiotensin Ii Receptor Blockers (Arbs), Plain	0.029	1.877E-06	5.727
Cardiovascular System	C10A_Lipid Modifying Agents, Plain	0.046	3.560E-14	13.449
Genito Urinary System and Sex Hormones	G03A_Hormonal Contraceptives for Systemic Use	-0.024	7.266E-05	4.139
Genito Urinary System and Sex Hormones	G03C_Estrogens	-0.027	6.733E-06	5.172
Genito Urinary System and Sex Hormones	G04C_Drugs Used in Benign Prostatic Hypertrophy	0.032	1.029E-07	6.988
Systemic Hormonal Preparations, Excl. Sex Hormones and Insulins	H03A_Thyroid Preparations	-0.028	5.169E-06	5.287
Musculo-Skeletal System	M04A_Antigout Preparations	0.022	3.133E-04	3.504
Nervous System	N02B_Other Analgesics and Antipyretics	-0.042	5.356E-12	11.271
Nervous System	N06A_Antidepressants	-0.031	3.507E-07	6.455
Sensory Organs	S01E_Antiglaucoma Preparations and Miotics	0.022	2.681E-04	3.572

Supplementary Table 3. Phenotypic associations of the ABCD cohort

**Subtype 1**				
**cat_id**	**phe_name**	**r_value**	**p_value**	**-logp_value**
Demographics	('interview_age', 'baseline_year_1_arm_1')	0.088	1.641E-19	18.785
Demographics	('interview_age', 'screener_arm_1')	0.088	1.641E-19	18.785
Demographics	('demo_prnt_age_v2_l', 'baseline_year_1_arm_1')	0.058	1.848E-09	8.733
Demographics	('demo_brthdat_v2', 'baseline_year_1_arm_1')	0.079	3.546E-16	15.450
Demographics	('demo_prnt_age_v2', 'baseline_year_1_arm_1')	0.060	6.557E-10	9.183
Demographics	('demo_ed_v2', 'baseline_year_1_arm_1')_4.0	-0.049	3.860E-07	6.413
Demographics	('demo_ed_v2', 'baseline_year_1_arm_1')_5.0	0.070	7.077E-13	12.150
Demographics	('demo_race_a_p___10', 'baseline_year_1_arm_1')_0.0	-0.090	2.179E-20	19.662
Demographics	('demo_race_a_p___10', 'baseline_year_1_arm_1')_1.0	0.090	2.179E-20	19.662
Demographics	('demo_race_a_p___11', 'baseline_year_1_arm_1')_0.0	0.094	3.259E-22	21.487
Demographics	('demo_race_a_p___11', 'baseline_year_1_arm_1')_1.0	-0.094	3.259E-22	21.487
Demographics	('demo_prnt_race_a_v2___10', 'baseline_year_1_arm_1')_0.0	-0.091	1.211E-20	19.917
Demographics	('demo_prnt_race_a_v2___10', 'baseline_year_1_arm_1')_1.0	0.091	1.211E-20	19.917
Demographics	('demo_prnt_race_a_v2___11', 'baseline_year_1_arm_1')_0.0	0.085	2.483E-18	17.605
Demographics	('demo_prnt_race_a_v2___11', 'baseline_year_1_arm_1')_1.0	-0.085	2.483E-18	17.605
Demographics	('demo_prnt_marital_v2', 'baseline_year_1_arm_1')_1.0	0.046	2.267E-06	5.645
Demographics	('demo_comb_income_v2', 'baseline_year_1_arm_1')_2.0	-0.049	5.663E-07	6.247
Demographics	('demo_fam_exp3_v2', 'baseline_year_1_arm_1')_0.0	0.047	1.703E-06	5.769

Demographics	('demo_fam_exp3_v2', 'baseline_year_1_arm_1')_1.0	-0.047	1.703E-06	5.769
Demographics	('fam_roster_2c_v2', 'baseline_year_1_arm_1')_1.0	0.048	7.875E-07	6.104
Socioeconomic	('ethnicity', '')_1.0	0.100	5.356E-25	24.271
Socioeconomic	('ethnicity', '')_2.0	-0.080	1.359E-16	15.867
Socioeconomic	('genetic_af_african', 'baseline_year_1_arm_1')	-0.091	4.813E-21	20.318
Socioeconomic	('genetic_af_european', 'baseline_year_1_arm_1')	0.111	2.039E-30	29.691
Socioeconomic	('genetic_af_east_asian', 'baseline_year_1_arm_1')	-0.045	4.280E-06	5.369
Socioeconomic	('reshist_addr1_adi_edu_h', 'baseline_year_1_arm_1')	0.046	2.228E-06	5.652
Socioeconomic	('reshist_addr1_adi_income', 'baseline_year_1_arm_1')	0.059	1.022E-09	8.991
Socioeconomic	('reshist_addr1_adi_in_dis', 'baseline_year_1_arm_1')	-0.054	3.052E-08	7.515
Socioeconomic	('reshist_addr1_adi_home_o', 'baseline_year_1_arm_1')	0.048	6.767E-07	6.170
Socioeconomic	('reshist_addr1_adi_unemp', 'baseline_year_1_arm_1')	-0.057	3.821E-09	8.418
Socioeconomic	('reshist_addr1_adi_pov', 'baseline_year_1_arm_1')	-0.063	6.951E-11	10.158
Socioeconomic	('reshist_addr1_adi_b138', 'baseline_year_1_arm_1')	-0.056	7.674E-09	8.115
Socioeconomic	('reshist_addr1_adi_sp', 'baseline_year_1_arm_1')	-0.071	2.500E-13	12.602
Socioeconomic	('reshist_addr1_adi_ncar', 'baseline_year_1_arm_1')	-0.046	2.075E-06	5.683
Socioeconomic	('reshist_addr1_adi_wsum', 'baseline_year_1_arm_1')	-0.050	2.945E-07	6.531
Socioeconomic	('reshist_addr1_adi_perc', 'baseline_year_1_arm_1')	-0.066	8.528E-12	11.069
Socioeconomic	('reshist_addr1_leadrisk_poverty', 'baseline_year_1_arm_1')	-0.059	1.536E-09	8.813
Socioeconomic	('reshist_addr1_coi_z_coi_nat', 'baseline_year_1_arm_1')	0.065	1.924E-11	10.716
Socioeconomic	('reshist_addr1_coi_c5_ed_nat', 'baseline_year_1_arm_1')	0.043	7.897E-06	5.103
Socioeconomic	('reshist_addr1_coi_c5_he_nat', 'baseline_year_1_arm_1')	0.052	9.647E-08	7.016
Socioeconomic	('reshist_addr1_coi_c5_se_nat', 'baseline_year_1_arm_1')	0.066	1.130E-11	10.947
Socioeconomic	('reshist_addr1_coi_c5_coi_nat', 'baseline_year_1_arm_1')	0.076	5.627E-15	14.250
Socioeconomic	('reshist_addr1_coi_r_ed_nat', 'baseline_year_1_arm_1')	0.053	4.299E-08	7.367
Socioeconomic	('reshist_addr1_coi_r_he_nat', 'baseline_year_1_arm_1')	0.050	2.856E-07	6.544
Socioeconomic	('reshist_addr1_coi_r_se_nat', 'baseline_year_1_arm_1')	0.070	6.237E-13	12.205
Socioeconomic	('reshist_addr1_coi_r_coi_nat', 'baseline_year_1_arm_1')	0.065	2.109E-11	10.676
Socioeconomic	('reshist_addr1_coi_c5_ed_stt', 'baseline_year_1_arm_1')	0.048	6.685E-07	6.175
Socioeconomic	('reshist_addr1_coi_c5_he_stt', 'baseline_year_1_arm_1')	0.062	1.468E-10	9.833
Socioeconomic	('reshist_addr1_coi_c5_se_stt', 'baseline_year_1_arm_1')	0.074	2.588E-14	13.587
Socioeconomic	('reshist_addr1_coi_c5_coi_stt', 'baseline_year_1_arm_1')	0.067	5.281E-12	11.277
Socioeconomic	('reshist_addr1_coi_r_ed_stt', 'baseline_year_1_arm_1')	0.046	1.801E-06	5.745
Socioeconomic	('reshist_addr1_coi_r_he_stt', 'baseline_year_1_arm_1')	0.065	1.847E-11	10.733
Socioeconomic	('reshist_addr1_coi_r_se_stt', 'baseline_year_1_arm_1')	0.076	4.028E-15	14.395
Socioeconomic	('reshist_addr1_coi_r_coi_stt', 'baseline_year_1_arm_1')	0.069	1.767E-12	11.753

Socioeconomic	('reshist_addr1_coi_ed_attain', 'baseline_year_1_arm_1')	0.048	6.642E-07	6.178
Socioeconomic	('reshist_addr1_coi_ed_schpov', 'baseline_year_1_arm_1')	-0.050	2.406E-07	6.619
Socioeconomic	('reshist_addr1_coi_se_povrate', 'baseline_year_1_arm_1')	-0.061	4.236E-10	9.373
Socioeconomic	('reshist_addr1_coi_se_public', 'baseline_year_1_arm_1')	-0.059	9.653E-10	9.015
Socioeconomic	('reshist_addr1_coi_se_home', 'baseline_year_1_arm_1')	0.046	2.286E-06	5.641
Socioeconomic	('reshist_addr1_coi_se_occ', 'baseline_year_1_arm_1')	0.048	8.656E-07	6.063
Socioeconomic	('reshist_addr1_coi_se_mhe', 'baseline_year_1_arm_1')	0.051	1.923E-07	6.716
Socioeconomic	('reshist_addr1_coi_se_emprat', 'baseline_year_1_arm_1')	0.050	3.200E-07	6.495
Socioeconomic	('reshist_addr1_coi_se_single', 'baseline_year_1_arm_1')	-0.073	6.046E-14	13.219
Socioeconomic	('reshist_addr1_coi_zed_attain', 'baseline_year_1_arm_1')	0.052	1.123E-07	6.950
Socioeconomic	('reshist_addr1_coi_zed_schpov', 'baseline_year_1_arm_1')	0.054	2.171E-08	7.663
Socioeconomic	('reshist_addr1_coi_zhe_hlthins', 'baseline_year_1_arm_1')	0.043	8.529E-06	5.069
Socioeconomic	('reshist_addr1_coi_zhe_vacancy', 'baseline_year_1_arm_1')	0.044	6.400E-06	5.194
Socioeconomic	('reshist_addr1_coi_zse_povrate', 'baseline_year_1_arm_1')	0.055	1.532E-08	7.815
Socioeconomic	('reshist_addr1_coi_zse_public', 'baseline_year_1_arm_1')	0.060	6.120E-10	9.213
Socioeconomic	('reshist_addr1_coi_zse_occ', 'baseline_year_1_arm_1')	0.052	1.196E-07	6.922
Socioeconomic	('reshist_addr1_coi_zse_mhe', 'baseline_year_1_arm_1')	0.056	8.542E-09	8.068
Socioeconomic	('reshist_addr1_coi_zse_emprat', 'baseline_year_1_arm_1')	0.055	2.065E-08	7.685
Socioeconomic	('reshist_addr1_coi_zse_single', 'baseline_year_1_arm_1')	0.074	1.874E-14	13.727
Socioeconomic	('reshist_addr1_coi_zse_ecres', 'baseline_year_1_arm_1')	0.061	2.856E-10	9.544
Socioeconomic	('reshist_addr1_svi_tot_20142018', 'baseline_year_1_arm_1')	-0.065	3.158E-11	10.501
Socioeconomic	('reshist_addr1_svi_pov_20142018', 'baseline_year_1_arm_1')	-0.064	4.043E-11	10.393
Socioeconomic	('reshist_addr1_svi_emp_20142018', 'baseline_year_1_arm_1')	-0.052	7.394E-08	7.131
Socioeconomic	('reshist_addr1_svi_cap_20142018', 'baseline_year_1_arm_1')	-0.061	4.384E-10	9.358
Socioeconomic	('reshist_addr1_svi_hs_20142018', 'baseline_year_1_arm_1')	-0.050	2.577E-07	6.589
Socioeconomic	('reshist_addr1_svi_sin_20142018', 'baseline_year_1_arm_1')	-0.062	1.603E-10	9.795
Socioeconomic	('reshist_addr1_svi_min_20142018', 'baseline_year_1_arm_1')	-0.065	1.720E-11	10.764
Socioeconomic	('reshist_addr1_svi_th1_20142018', 'baseline_year_1_arm_1')	-0.068	2.203E-12	11.657
Socioeconomic	('reshist_addr1_svi_th2_20142018', 'baseline_year_1_arm_1')	-0.055	1.244E-08	7.905
Socioeconomic	('reshist_addr1_svi_th3_20142018', 'baseline_year_1_arm_1')	-0.048	8.067E-07	6.093
Socioeconomic	('reshist_addr1_opat_kfrpp_p1', 'baseline_year_1_arm_1')	0.055	1.333E-08	7.875
Socioeconomic	('reshist_addr1_opat_kfrpp_p25', 'baseline_year_1_arm_1')	0.063	7.300E-11	10.137
Socioeconomic	('reshist_addr1_opat_kfrpp_p50', 'baseline_year_1_arm_1')	0.068	3.077E-12	11.512
Socioeconomic	('reshist_addr1_opat_kfrpp_p75', 'baseline_year_1_arm_1')	0.071	3.274E-13	12.485
Socioeconomic	('reshist_addr1_opat_kfrpp_p100', 'baseline_year_1_arm_1')	0.053	5.544E-08	7.256

Socioeconomic	('reshist_addr1_opat_kfrpp_avg', 'baseline_year_1_arm_1')	0.068	2.513E-12	11.600
Socioeconomic	('reshist_addr1_coi_z_ed_nat', 'baseline_year_1_arm_1')	0.051	1.852E-07	6.732
Socioeconomic	('reshist_addr1_coi_z_he_nat', 'baseline_year_1_arm_1')	0.056	8.523E-09	8.069
Socioeconomic	('reshist_addr1_coi_z_se_nat', 'baseline_year_1_arm_1')	0.069	1.069E-12	11.971
Socioeconomic	('rel_relationship', 'baseline_year_1_arm_1')_2.0	0.049	5.326E-07	6.274
Summary	('pps_y_ss_severity_score', 'baseline_year_1_arm_1')	-0.045	3.827E-06	5.417
Med History	('fhx_3hb_p', 'baseline_year_1_arm_1')	-0.044	6.173E-06	5.210
Task Based	('lmt_scr_num_correct', 'baseline_year_1_arm_1')	0.063	9.718E-11	10.012
Task Based	('lmt_scr_num_wrong', 'baseline_year_1_arm_1')	-0.049	3.778E-07	6.423
Task Based	('tfmri_nb_all_beh_ctotal_nt', 'baseline_year_1_arm_1')	0.078	1.426E-15	14.846
Task Based	('tfmri_nb_all_beh_c2b_nt', 'baseline_year_1_arm_1')	0.076	6.828E-15	14.166
Task Based	('tfmri_nb_r1_beh_c2b_nt', 'baseline_year_1_arm_1')	0.072	1.579E-13	12.802
Task Based	('tfmri_nb_r2_beh_c2b_nt', 'baseline_year_1_arm_1')	0.077	1.692E-15	14.772
Task Based	('tfmri_nb_all_beh_cpf_nt', 'baseline_year_1_arm_1')	0.078	8.593E-16	15.066
Task Based	('tfmri_nb_r1_beh_cpf_nt', 'baseline_year_1_arm_1')	0.058	2.346E-09	8.630
Task Based	('tfmri_nb_r2_beh_cpf_nt', 'baseline_year_1_arm_1')	0.077	2.532E-15	14.596
Task Based	('tfmri_nb_all_beh_c2bpf_nt', 'baseline_year_1_arm_1')	0.065	3.235E-11	10.490
Task Based	('tfmri_nb_r2_beh_c2bpf_nt', 'baseline_year_1_arm_1')	0.071	2.293E-13	12.640
Task Based	('tfmri_nb_all_beh_c2bpfnl_nt', 'baseline_year_1_arm_1')	0.046	2.890E-06	5.539
Task Based	('tfmri_nb_all_beh_cnf_nt', 'baseline_year_1_arm_1')	0.073	4.481E-14	13.349
Task Based	('tfmri_nb_r1_beh_cnf_nt', 'baseline_year_1_arm_1')	0.063	1.080E-10	9.967
Task Based	('tfmri_nb_r2_beh_cnf_nt', 'baseline_year_1_arm_1')	0.073	7.095E-14	13.149
Task Based	('tfmri_nb_all_beh_c2bnf_nt', 'baseline_year_1_arm_1')	0.074	3.008E-14	13.522
Task Based	('tfmri_nb_r1_beh_c2bnf_nt', 'baseline_year_1_arm_1')	0.057	3.850E-09	8.415
Task Based	('tfmri_nb_r2_beh_c2bnf_nt', 'baseline_year_1_arm_1')	0.065	2.874E-11	10.542
Task Based	('tfmri_nb_all_beh_c2bnfnl_nt', 'baseline_year_1_arm_1')	0.057	5.659E-09	8.247
Task Based	('tfmri_nb_all_beh_cngf_nt', 'baseline_year_1_arm_1')	0.069	1.185E-12	11.926
Task Based	('tfmri_nb_r1_beh_cngf_nt', 'baseline_year_1_arm_1')	0.066	1.584E-11	10.800
Task Based	('tfmri_nb_r2_beh_cngf_nt', 'baseline_year_1_arm_1')	0.062	1.402E-10	9.853
Task Based	('tfmri_nb_all_beh_c2bngf_nt', 'baseline_year_1_arm_1')	0.065	2.764E-11	10.558
Task Based	('tfmri_nb_r1_beh_c2bngf_nt', 'baseline_year_1_arm_1')	0.062	1.766E-10	9.753
Task Based	('tfmri_nb_r2_beh_c2bngf_nt', 'baseline_year_1_arm_1')	0.055	1.493E-08	7.826
Task Based	('tfmri_nb_all_beh_cplace_nt', 'baseline_year_1_arm_1')	0.075	1.409E-14	13.851
Task Based	('tfmri_nb_r1_beh_cplace_nt', 'baseline_year_1_arm_1')	0.060	8.785E-10	9.056
Task Based	('tfmri_nb_r2_beh_cplace_nt', 'baseline_year_1_arm_1')	0.068	3.881E-12	11.411

Task Based	('tfmri_nb_all_beh_c2bp_nt', 'baseline_year_1_arm_1')	0.067	7.532E-12	11.123
Task Based	('tfmri_nb_r1_beh_c2bp_nt', 'baseline_year_1_arm_1')	0.051	1.974E-07	6.705
Task Based	('tfmri_nb_r2_beh_c2bp_nt', 'baseline_year_1_arm_1')	0.062	1.452E-10	9.838
Task Based	('tfmri_nb_all_beh_c2bpnl_nt', 'baseline_year_1_arm_1')	0.051	1.464E-07	6.835
Task Based	('tfmri_nb_all_beh_c0b_nt', 'baseline_year_1_arm_1')	0.072	1.247E-13	12.904
Task Based	('tfmri_nb_r1_beh_c0b_nt', 'baseline_year_1_arm_1')	0.066	1.540E-11	10.813
Task Based	('tfmri_nb_r2_beh_c0b_nt', 'baseline_year_1_arm_1')	0.069	1.710E-12	11.767
Task Based	('tfmri_nb_all_beh_c0bpf_nt', 'baseline_year_1_arm_1')	0.069	1.703E-12	11.769
Task Based	('tfmri_nb_r1_beh_c0bpf_nt', 'baseline_year_1_arm_1')	0.060	6.135E-10	9.212
Task Based	('tfmri_nb_r2_beh_c0bpf_nt', 'baseline_year_1_arm_1')	0.065	1.998E-11	10.699
Task Based	('tfmri_nb_all_beh_c0bpfnl_nt', 'baseline_year_1_arm_1')	0.047	1.413E-06	5.850
Task Based	('tfmri_nb_all_beh_c0bnf_nt', 'baseline_year_1_arm_1')	0.059	1.299E-09	8.887
Task Based	('tfmri_nb_r1_beh_c0bnf_nt', 'baseline_year_1_arm_1')	0.047	1.432E-06	5.844
Task Based	('tfmri_nb_r2_beh_c0bnf_nt', 'baseline_year_1_arm_1')	0.058	2.181E-09	8.661
Task Based	('tfmri_nb_all_beh_c0bnfnl_nt', 'baseline_year_1_arm_1')	0.049	5.664E-07	6.247
Task Based	('tfmri_nb_all_beh_c0bngf_nt', 'baseline_year_1_arm_1')	0.062	2.542E-10	9.595
Task Based	('tfmri_nb_r1_beh_c0bngf_nt', 'baseline_year_1_arm_1')	0.048	1.008E-06	5.996
Task Based	('tfmri_nb_r2_beh_c0bngf_nt', 'baseline_year_1_arm_1')	0.049	4.658E-07	6.332
Task Based	('tfmri_nb_all_beh_c0bngfnl_nt', 'baseline_year_1_arm_1')	0.048	6.714E-07	6.173
Task Based	('tfmri_nb_all_beh_c0bp_nt', 'baseline_year_1_arm_1')	0.059	1.171E-09	8.931
Task Based	('tfmri_nb_r1_beh_c0bp_nt', 'baseline_year_1_arm_1')	0.050	2.142E-07	6.669
Task Based	('tfmri_nb_r2_beh_c0bp_nt', 'baseline_year_1_arm_1')	0.053	5.699E-08	7.244
Task Based	('tfmri_nb_all_beh_c0bpnl_nt', 'baseline_year_1_arm_1')	0.053	4.156E-08	7.381
Task Based	('lmt_scr_perc_correct', 'baseline_year_1_arm_1')	0.058	1.956E-09	8.709
Task Based	('lmt_scr_perc_wrong', 'baseline_year_1_arm_1')	-0.049	4.211E-07	6.376
Task Based	('tfmri_nb_all_beh_ctotal_rate', 'baseline_year_1_arm_1')	0.080	1.274E-16	15.895
Task Based	('tfmri_nb_all_beh_c2b_rate', 'baseline_year_1_arm_1')	0.078	1.284E-15	14.891
Task Based	('tfmri_nb_r1_beh_c2b_rate', 'baseline_year_1_arm_1')	0.070	8.725E-13	12.059
Task Based	('tfmri_nb_r2_beh_c2b_rate', 'baseline_year_1_arm_1')	0.073	6.785E-14	13.168
Task Based	('tfmri_nb_all_beh_cpf_rate', 'baseline_year_1_arm_1')	0.079	6.361E-16	15.196
Task Based	('tfmri_nb_all_beh_cpf_stdrt', 'baseline_year_1_arm_1')	-0.046	2.505E-06	5.601
Task Based	('tfmri_nb_r1_beh_cpf_rate', 'baseline_year_1_arm_1')	0.058	2.189E-09	8.660
Task Based	('tfmri_nb_r2_beh_cpf_rate', 'baseline_year_1_arm_1')	0.079	6.379E-16	15.195
Task Based	('tfmri_nb_all_beh_c2bpf_rate', 'baseline_year_1_arm_1')	0.064	6.262E-11	10.203
Task Based	('tfmri_nb_r2_beh_c2bpf_rate', 'baseline_year_1_arm_1')	0.068	2.374E-12	11.624

Task Based	('tfmri_nb_all_beh_c2bpft_rate', 'baseline_year_1_arm_1')	0.044	6.166E-06	5.210
Task Based	('tfmri_nb_all_beh_c2bpfl_rate', 'baseline_year_1_arm_1')	0.044	5.687E-06	5.245
Task Based	('tfmri_nb_r2_beh_c2bpfl_rate', 'baseline_year_1_arm_1')	0.055	1.576E-08	7.802
Task Based	('tfmri_nb_all_beh_c2bpfnl_rate', 'baseline_year_1_arm_1')	0.049	3.907E-07	6.408
Task Based	('tfmri_nb_r2_beh_c2bpfnl_rate', 'baseline_year_1_arm_1')	0.055	1.916E-08	7.718
Task Based	('tfmri_nb_all_beh_cnf_rate', 'baseline_year_1_arm_1')	0.074	2.648E-14	13.577
Task Based	('tfmri_nb_r1_beh_cnf_rate', 'baseline_year_1_arm_1')	0.062	1.867E-10	9.729
Task Based	('tfmri_nb_r2_beh_cnf_rate', 'baseline_year_1_arm_1')	0.075	1.049E-14	13.979
Task Based	('tfmri_nb_all_beh_c2bnf_rate', 'baseline_year_1_arm_1')	0.073	6.010E-14	13.221
Task Based	('tfmri_nb_r1_beh_c2bnf_rate', 'baseline_year_1_arm_1')	0.061	3.973E-10	9.401
Task Based	('tfmri_nb_r2_beh_c2bnf_rate', 'baseline_year_1_arm_1')	0.067	5.646E-12	11.248
Task Based	('tfmri_nb_all_beh_c2bnfl_rate', 'baseline_year_1_arm_1')	0.058	2.196E-09	8.658
Task Based	('tfmri_nb_r2_beh_c2bnfl_rate', 'baseline_year_1_arm_1')	0.051	1.488E-07	6.827
Task Based	('tfmri_nb_all_beh_c2bnfnl_rate', 'baseline_year_1_arm_1')	0.061	2.963E-10	9.528
Task Based	('tfmri_nb_r1_beh_c2bnfnl_rate', 'baseline_year_1_arm_1')	0.051	2.024E-07	6.694
Task Based	('tfmri_nb_r2_beh_c2bnfnl_rate', 'baseline_year_1_arm_1')	0.049	4.035E-07	6.394
Task Based	('tfmri_nb_all_beh_cngf_rate', 'baseline_year_1_arm_1')	0.071	3.783E-13	12.422
Task Based	('tfmri_nb_r1_beh_cngf_rate', 'baseline_year_1_arm_1')	0.067	5.398E-12	11.268
Task Based	('tfmri_nb_r2_beh_cngf_rate', 'baseline_year_1_arm_1')	0.062	1.319E-10	9.880
Task Based	('tfmri_nb_all_beh_c2bngf_rate', 'baseline_year_1_arm_1')	0.067	4.095E-12	11.388
Task Based	('tfmri_nb_r1_beh_c2bngf_rate', 'baseline_year_1_arm_1')	0.062	1.849E-10	9.733
Task Based	('tfmri_nb_r2_beh_c2bngf_rate', 'baseline_year_1_arm_1')	0.052	1.077E-07	6.968
Task Based	('tfmri_nb_all_beh_c2bngft_rate', 'baseline_year_1_arm_1')	0.053	6.344E-08	7.198
Task Based	('tfmri_nb_all_beh_c2bngfl_rate', 'baseline_year_1_arm_1')	0.054	3.350E-08	7.475
Task Based	('tfmri_nb_r2_beh_c2bngfl_rate', 'baseline_year_1_arm_1')	0.044	6.192E-06	5.208
Task Based	('tfmri_nb_all_beh_c2bngfnl_rate', 'baseline_year_1_arm_1')	0.045	4.679E-06	5.330
Task Based	('tfmri_nb_r1_beh_c2bngfnl_rate', 'baseline_year_1_arm_1')	0.046	2.357E-06	5.628
Task Based	('tfmri_nb_all_beh_cplace_rate', 'baseline_year_1_arm_1')	0.073	7.281E-14	13.138
Task Based	('tfmri_nb_r1_beh_cplace_rate', 'baseline_year_1_arm_1')	0.060	7.500E-10	9.125
Task Based	('tfmri_nb_r2_beh_cplace_rate', 'baseline_year_1_arm_1')	0.064	4.967E-11	10.304
Task Based	('tfmri_nb_all_beh_c2bp_rate', 'baseline_year_1_arm_1')	0.064	6.131E-11	10.212
Task Based	('tfmri_nb_r1_beh_c2bp_rate', 'baseline_year_1_arm_1')	0.047	1.446E-06	5.840
Task Based	('tfmri_nb_r2_beh_c2bp_rate', 'baseline_year_1_arm_1')	0.059	1.119E-09	8.951
Task Based	('tfmri_nb_all_beh_c2bpl_rate', 'baseline_year_1_arm_1')	0.054	2.605E-08	7.584
Task Based	('tfmri_nb_r2_beh_c2bpl_rate', 'baseline_year_1_arm_1')	0.046	2.264E-06	5.645

Task Based	('tfmri_nb_all_beh_c2bpnl_rate', 'baseline_year_1_arm_1')	0.051	1.236E-07	6.908
Task Based	('tfmri_nb_r2_beh_c2bpnl_rate', 'baseline_year_1_arm_1')	0.044	5.225E-06	5.282
Task Based	('tfmri_nb_all_beh_c0b_rate', 'baseline_year_1_arm_1')	0.073	7.458E-14	13.127
Task Based	('tfmri_nb_all_beh_c0b_stdrt', 'baseline_year_1_arm_1')	-0.063	7.465E-11	10.127
Task Based	('tfmri_nb_r1_beh_c0b_rate', 'baseline_year_1_arm_1')	0.065	2.452E-11	10.610
Task Based	('tfmri_nb_r1_beh_c0b_stdrt', 'baseline_year_1_arm_1')	-0.052	8.036E-08	7.095
Task Based	('tfmri_nb_r2_beh_c0b_rate', 'baseline_year_1_arm_1')	0.073	8.281E-14	13.082
Task Based	('tfmri_nb_r2_beh_c0b_mrt', 'baseline_year_1_arm_1')	-0.047	1.746E-06	5.758
Task Based	('tfmri_nb_r2_beh_c0b_stdrt', 'baseline_year_1_arm_1')	-0.062	1.531E-10	9.815
Task Based	('tfmri_nb_all_beh_c0bpf_rate', 'baseline_year_1_arm_1')	0.070	6.473E-13	12.189
Task Based	('tfmri_nb_all_beh_c0bpf_stdrt', 'baseline_year_1_arm_1')	-0.050	3.621E-07	6.441
Task Based	('tfmri_nb_r1_beh_c0bpf_rate', 'baseline_year_1_arm_1')	0.059	1.395E-09	8.855
Task Based	('tfmri_nb_r2_beh_c0bpf_rate', 'baseline_year_1_arm_1')	0.064	3.635E-11	10.440
Task Based	('tfmri_nb_all_beh_c0bpft_rate', 'baseline_year_1_arm_1')	0.054	3.791E-08	7.421
Task Based	('tfmri_nb_all_beh_c0bpfl_rate', 'baseline_year_1_arm_1')	0.069	9.153E-13	12.038
Task Based	('tfmri_nb_r1_beh_c0bpfl_rate', 'baseline_year_1_arm_1')	0.059	1.244E-09	8.905
Task Based	('tfmri_nb_r2_beh_c0bpfl_rate', 'baseline_year_1_arm_1')	0.065	1.705E-11	10.768
Task Based	('tfmri_nb_all_beh_c0bpfnl_rate', 'baseline_year_1_arm_1')	0.050	3.344E-07	6.476
Task Based	('tfmri_nb_all_beh_c0bnf_rate', 'baseline_year_1_arm_1')	0.060	8.625E-10	9.064
Task Based	('tfmri_nb_all_beh_c0bnf_mrt', 'baseline_year_1_arm_1')	-0.044	5.779E-06	5.238
Task Based	('tfmri_nb_all_beh_c0bnf_stdrt', 'baseline_year_1_arm_1')	-0.045	4.192E-06	5.378
Task Based	('tfmri_nb_r1_beh_c0bnf_rate', 'baseline_year_1_arm_1')	0.049	5.741E-07	6.241
Task Based	('tfmri_nb_r2_beh_c0bnf_rate', 'baseline_year_1_arm_1')	0.058	1.865E-09	8.729
Task Based	('tfmri_nb_r2_beh_c0bnf_mrt', 'baseline_year_1_arm_1')	-0.044	6.746E-06	5.171
Task Based	('tfmri_nb_all_beh_c0bnft_rate', 'baseline_year_1_arm_1')	0.044	6.017E-06	5.221
Task Based	('tfmri_nb_all_beh_c0bnfl_rate', 'baseline_year_1_arm_1')	0.055	1.951E-08	7.710
Task Based	('tfmri_nb_r2_beh_c0bnfl_rate', 'baseline_year_1_arm_1')	0.058	2.543E-09	8.595
Task Based	('tfmri_nb_all_beh_c0bnfnl_rate', 'baseline_year_1_arm_1')	0.044	7.392E-06	5.131
Task Based	('tfmri_nb_all_beh_c0bngf_rate', 'baseline_year_1_arm_1')	0.057	3.700E-09	8.432
Task Based	('tfmri_nb_r1_beh_c0bngf_rate', 'baseline_year_1_arm_1')	0.047	1.118E-06	5.952
Task Based	('tfmri_nb_r2_beh_c0bngf_rate', 'baseline_year_1_arm_1')	0.051	1.732E-07	6.761
Task Based	('tfmri_nb_all_beh_c0bngfl_rate', 'baseline_year_1_arm_1')	0.053	3.890E-08	7.410
Task Based	('tfmri_nb_r2_beh_c0bngfl_rate', 'baseline_year_1_arm_1')	0.044	5.597E-06	5.252
Task Based	('tfmri_nb_all_beh_c0bngfnl_rate', 'baseline_year_1_arm_1')	0.049	4.428E-07	6.354
Task Based	('tfmri_nb_all_beh_c0bp_rate', 'baseline_year_1_arm_1')	0.060	8.998E-10	9.046

Task Based	('tfmri_nb_r1_beh_c0bp_rate', 'baseline_year_1_arm_1')	0.050	2.189E-07	6.660
Task Based	('tfmri_nb_r2_beh_c0bp_rate', 'baseline_year_1_arm_1')	0.051	1.331E-07	6.876
Task Based	('tfmri_nb_all_beh_c0bpt_rate', 'baseline_year_1_arm_1')	0.050	2.373E-07	6.625
Task Based	('tfmri_nb_r1_beh_c0bpt_rate', 'baseline_year_1_arm_1')	0.044	5.306E-06	5.275
Task Based	('tfmri_nb_all_beh_c0bpl_rate', 'baseline_year_1_arm_1')	0.046	1.783E-06	5.749
Task Based	('tfmri_nb_all_beh_c0bpnl_rate', 'baseline_year_1_arm_1')	0.049	4.733E-07	6.325
Task Based	('tfmri_nb_r2_beh_c0bpnl_rate', 'baseline_year_1_arm_1')	0.045	3.580E-06	5.446
Task Based	('tfmri_nback_beh_performflag', 'baseline_year_1_arm_1')_0.0	-0.056	7.941E-09	8.100
Task Based	('tfmri_nback_beh_performflag', 'baseline_year_1_arm_1')_1.0	0.056	7.941E-09	8.100
Task Based	('lmt_scr_correct_num_stimtype3', 'baseline_year_1_arm_1')_0.0	-0.050	2.638E-07	6.579
Task Based	('lmt_scr_correct_num_stimtype4', 'baseline_year_1_arm_1')_0.0	-0.056	8.662E-09	8.062
Task Based	('lmt_scr_correct_num_stimtype4', 'baseline_year_1_arm_1')_4.0	0.047	1.123E-06	5.950
Task Based	('lmt_scr_wrong_num_stimtype3', 'baseline_year_1_arm_1')_0.0	0.051	1.882E-07	6.725
Task Based	('lmt_scr_wrong_num_stimtype3', 'baseline_year_1_arm_1')_3.0	-0.050	2.593E-07	6.586
Task Based	('lmt_scr_wrong_num_stimtype4', 'baseline_year_1_arm_1')_0.0	0.045	4.078E-06	5.390
Task Based	('lmt_scr_wrong_num_stimtype4', 'baseline_year_1_arm_1')_3.0	-0.048	8.998E-07	6.046
Task Based	('tfmri_nb_r2_beh_c2bpfl_nt', 'baseline_year_1_arm_1')_2.0	0.059	1.617E-09	8.791
Task Based	('tfmri_nb_all_beh_c2bnfl_nt', 'baseline_year_1_arm_1')_4.0	0.049	3.750E-07	6.426
Task Based	('tfmri_nb_r1_beh_c2bnfl_nt', 'baseline_year_1_arm_1')_2.0	0.047	1.161E-06	5.935
Task Based	('tfmri_nb_r2_beh_c2bnfl_nt', 'baseline_year_1_arm_1')_2.0	0.051	1.632E-07	6.787
Task Based	('tfmri_nb_r1_beh_c2bnfnl_nt', 'baseline_year_1_arm_1')_6.0	0.052	1.040E-07	6.983
Task Based	('tfmri_nb_r1_beh_c2bngfnl_nt', 'baseline_year_1_arm_1')_3.0	-0.045	3.929E-06	5.406
Task Based	('tfmri_nb_r2_beh_c2bpl_nt', 'baseline_year_1_arm_1')_2.0	0.048	7.695E-07	6.114
Task Based	('tfmri_nb_all_beh_c0bpfl_nt', 'baseline_year_1_arm_1')_6.0	0.074	2.828E-14	13.548
Task Based	('tfmri_nb_r1_beh_c0bpfl_nt', 'baseline_year_1_arm_1')_3.0	0.056	6.502E-09	8.187
Task Based	('tfmri_nb_r2_beh_c0bpfl_nt', 'baseline_year_1_arm_1')_1.0	-0.046	2.438E-06	5.613
Task Based	('tfmri_nb_r2_beh_c0bpfl_nt', 'baseline_year_1_arm_1')_3.0	0.068	2.783E-12	11.556
Task Based	('tfmri_nb_r1_beh_c0bnft_nt', 'baseline_year_1_arm_1')_2.0	0.044	7.067E-06	5.151
Task Based	('tfmri_nb_r2_beh_c0bnfl_nt', 'baseline_year_1_arm_1')_3.0	0.053	6.730E-08	7.172
Task Based	('tfmri_nb_r2_beh_c0bngfnl_nt', 'baseline_year_1_arm_1')_5.0	0.044	5.991E-06	5.223
Task Based	('tfmri_nb_all_beh_c0bpt_nt', 'baseline_year_1_arm_1')_4.0	0.052	8.722E-08	7.059
Task Based	('tfmri_nb_r1_beh_c0bpt_nt', 'baseline_year_1_arm_1')_2.0	0.049	4.890E-07	6.311
Task Based	('tfmri_nb_r1_beh_c0bpl_nt', 'baseline_year_1_arm_1')_1.0	-0.046	2.770E-06	5.557
Cognitive	('pea_ravlt_sd_trial_ii_tc', 'baseline_year_1_arm_1')	0.047	1.223E-06	5.913
Cognitive	('pea_ravlt_sd_trial_iii_tc', 'baseline_year_1_arm_1')	0.045	4.443E-06	5.352

Cognitive	('pea_ravlt_sd_trial_iv_tc', 'baseline_year_1_arm_1')	0.049	3.690E-07	6.433
Cognitive	('pea_ravlt_sd_trial_v_tc', 'baseline_year_1_arm_1')	0.054	2.643E-08	7.578
Cognitive	('pea_ravlt_sd_trial_vi_tc', 'baseline_year_1_arm_1')	0.046	2.100E-06	5.678
Cognitive	('pea_ravlt_ld_trial_vii_tc', 'baseline_year_1_arm_1')	0.047	1.178E-06	5.929
Cognitive	('pea_wiscv_trs', 'baseline_year_1_arm_1')	0.061	2.605E-10	9.584
Cognitive	('pea_wiscv_tss', 'baseline_year_1_arm_1')	0.046	1.853E-06	5.732
Cognitive	('nihtbx_flanker_agecorrected', 'baseline_year_1_arm_1')_78.0	-0.043	8.480E-06	5.072
Cognitive	('nihtbx_flanker_agecorrected', 'baseline_year_1_arm_1')_92.0	0.055	1.568E-08	7.805
Cognitive	('nihtbx_cardsort_agecorrected', 'baseline_year_1_arm_1')_81.0	-0.057	4.096E-09	8.388
Cognitive	('nihtbx_picture_agecorrected', 'baseline_year_1_arm_1')_88.0	-0.047	1.605E-06	5.794
Behavior	('cbcl_q53_p', 'baseline_year_1_arm_1')_0.0	0.062	2.484E-10	9.605
Behavior	('cbcl_q53_p', 'baseline_year_1_arm_1')_1.0	-0.049	4.131E-07	6.384
Behavior	('cbcl_q55_p', 'baseline_year_1_arm_1')_0.0	0.070	5.951E-13	12.225
Behavior	('cbcl_q55_p', 'baseline_year_1_arm_1')_1.0	-0.050	2.285E-07	6.641
Behavior	('cbcl_q55_p', 'baseline_year_1_arm_1')_2.0	-0.048	8.812E-07	6.055
Activity	('sai_p_activities___1', 'baseline_year_1_arm_1')_0.0	-0.046	1.826E-06	5.738
Activity	('sai_p_activities___1', 'baseline_year_1_arm_1')_1.0	0.046	1.826E-06	5.738
Activity	('sai_p_activities___15', 'baseline_year_1_arm_1')_0.0	-0.053	3.900E-08	7.409
Activity	('sai_p_activities___15', 'baseline_year_1_arm_1')_1.0	0.053	3.900E-08	7.409
Activity	('sai_ss_base_nyr_p', 'baseline_year_1_arm_1')_0.0	-0.049	5.971E-07	6.224
Activity	('sai_ss_base_nmonth_p', 'baseline_year_1_arm_1')_0.0	-0.048	6.360E-07	6.197
Activity	('sai_ss_base_nmonth_p', 'baseline_year_1_arm_1')_4.0	0.050	2.440E-07	6.613
Activity	('sai_ss_base_perwk_p', 'baseline_year_1_arm_1')_0.0	-0.048	9.302E-07	6.031
Activity	('sai_ss_base_tspent_p', 'baseline_year_1_arm_1')_0.0	-0.048	6.819E-07	6.166
Activity	('sai_ss_soc_nyr_p', 'baseline_year_1_arm_1')_0.0	-0.054	2.251E-08	7.648
Activity	('sai_ss_soc_nmonth_p', 'baseline_year_1_arm_1')_0.0	-0.054	3.335E-08	7.477
Activity	('sai_ss_soc_perwk_p', 'baseline_year_1_arm_1')_0.0	-0.053	4.085E-08	7.389
Activity	('sai_ss_soc_tspent_p', 'baseline_year_1_arm_1')_0.0	-0.055	1.875E-08	7.727
Social Adjustment	('kbi_p_c_school_setting', 'baseline_year_1_arm_1')_3.0	0.044	6.610E-06	5.180
Social Adjustment	('kbi_p_how_well_c_school', 'baseline_year_1_arm_1')_1.0	0.044	6.287E-06	5.202
Social Adjustment	('mex_american10_p', 'baseline_year_1_arm_1')_5.0	-0.045	4.783E-06	5.320
Social Adjustment	('meim_ethnic_id_p', 'baseline_year_1_arm_1')_1.0	0.083	8.795E-18	17.056
Social Adjustment	('meim_ethnic_id_p', 'baseline_year_1_arm_1')_5.0	-0.075	1.432E-14	13.844
Substance Use	('tlfb_age_calc_inmonths', 'baseline_year_1_arm_1')	0.087	4.360E-19	18.361
Substance Use	('tlfb_tob', 'baseline_year_1_arm_1')_0.0	-0.043	8.469E-06	5.072

Substance Use	('tlfb_tob', 'baseline_year_1_arm_1')_1.0	0.043	8.469E-06	5.072
Substance Use	('tlfb_age', 'baseline_year_1_arm_1')_9.0	-0.082	4.679E-17	16.330
Substance Use	('tlfb_age', 'baseline_year_1_arm_1')_10.0	0.082	3.209E-17	16.494
Personality	('upps28_y', 'baseline_year_1_arm_1')_1.0	-0.044	5.508E-06	5.259
Personality	('upps36_y', 'baseline_year_1_arm_1')_4.0	-0.047	1.301E-06	5.886
Personality	('upps39_y', 'baseline_year_1_arm_1')_4.0	-0.044	6.699E-06	5.174
Questionnaire	('postscan_excited_1', 'baseline_year_1_arm_1')_5.0	-0.043	8.105E-06	5.091
Questionnaire	('scrn_twin_reg___2', 'screener_arm_1')_0.0	-0.046	2.569E-06	5.590
Questionnaire	('scrn_twin_reg___2', 'screener_arm_1')_1.0	0.046	2.569E-06	5.590
Questionnaire	('bisbas3_y', 'baseline_year_1_arm_1')_1.0	0.046	2.167E-06	5.664
Questionnaire	('screen7_wknd_y', 'baseline_year_1_arm_1')_4.0	-0.048	7.105E-07	6.148
Diagnostic	('ksads_1_188_p', 'baseline_year_1_arm_1')_0.0	-0.044	7.343E-06	5.134
Diagnostic	('ksads_1_188_p', 'baseline_year_1_arm_1')_1.0	0.044	7.343E-06	5.134
Diagnostic	('ksads_13_74_p', 'baseline_year_1_arm_1')_0.0	0.051	1.678E-07	6.775
Diagnostic	('ksads_13_74_p', 'baseline_year_1_arm_1')_1.0	-0.051	1.678E-07	6.775
		**Subtype 2**		
**cat_id**	**phe_name**	**r_value**	**p_value**	**-logp_value**
Demographics	('sex_official', '')_F	0.150	2.331E-54	53.632
Demographics	('sex_official', '')_M	-0.150	2.331E-54	53.632
Demographics	('interview_age', 'baseline_year_1_arm_1')	-0.072	1.216E-13	12.915
Demographics	('interview_age', 'screener_arm_1')	-0.072	1.216E-13	12.915
Demographics	('demo_brthdat_v2', 'baseline_year_1_arm_1')	-0.059	1.414E-09	8.850
Demographics	('demo_sex_v2', 'baseline_year_1_arm_1')_1.0	-0.150	1.752E-54	53.756
Demographics	('demo_sex_v2', 'baseline_year_1_arm_1')_2.0	0.150	2.998E-54	53.523
Demographics	('demo_gender_id_v2', 'baseline_year_1_arm_1')_1.0	-0.149	2.899E-53	52.538
Demographics	('demo_gender_id_v2', 'baseline_year_1_arm_1')_2.0	0.149	1.641E-53	52.785
Socioeconomic	('acs_raked_propensity_score', 'baseline_year_1_arm_1')	-0.045	4.331E-06	5.363
Socioeconomic	('genetic_af_american', 'baseline_year_1_arm_1')	-0.063	7.345E-11	10.134
Med History	('stq_y_ss_weekend', 'baseline_year_1_arm_1')	-0.047	1.148E-06	5.940
Med History	('medhx_2a', 'baseline_year_1_arm_1')_0.0	0.048	8.013E-07	6.096
Med History	('medhx_2a', 'baseline_year_1_arm_1')_1.0	-0.048	8.013E-07	6.096
Task Based	('tfmri_nb_all_beh_c2bpt_mrt', 'baseline_year_1_arm_1')	0.044	5.470E-06	5.262
Cognitive	('pea_wiscv_tss', 'baseline_year_1_arm_1')	0.046	1.856E-06	5.731
Behavior	('cbcl_scr_syn_withdep_t', 'baseline_year_1_arm_1')	-0.046	1.906E-06	5.720
Behavior	('cbcl_scr_syn_attention_r', 'baseline_year_1_arm_1')	-0.052	7.010E-08	7.154
Behavior	('cbcl_scr_dsm5_adhd_r', 'baseline_year_1_arm_1')	-0.055	1.633E-08	7.787
Behavior	('cbcl_q08_p', 'baseline_year_1_arm_1')_0.0	0.055	1.281E-08	7.893

Behavior	('cbcl_q08_p', 'baseline_year_1_arm_1')_2.0	-0.049	3.675E-07	6.435
Behavior	('cbcl_q10_p', 'baseline_year_1_arm_1')_0.0	0.056	9.345E-09	8.029
Behavior	('cbcl_q61_p', 'baseline_year_1_arm_1')_0.0	0.044	5.032E-06	5.298
Behavior	('cbcl_q78_p', 'baseline_year_1_arm_1')_0.0	0.047	1.468E-06	5.833
Activity	('sai_p_activities___0', 'baseline_year_1_arm_1')_0.0	-0.076	3.987E-15	14.399
Activity	('sai_p_activities___0', 'baseline_year_1_arm_1')_1.0	0.076	3.987E-15	14.399
Activity	('sai_p_activities___6', 'baseline_year_1_arm_1')_0.0	-0.065	2.001E-11	10.699
Activity	('sai_p_activities___6', 'baseline_year_1_arm_1')_1.0	0.065	2.001E-11	10.699
Activity	('sai_p_activities___23', 'baseline_year_1_arm_1')_0.0	-0.056	9.139E-09	8.039
Activity	('sai_p_activities___23', 'baseline_year_1_arm_1')_1.0	0.056	9.139E-09	8.039
Activity	('sai_ss_dance_nyr_p', 'baseline_year_1_arm_1')_0.0	-0.078	7.460E-16	15.127
Activity	('sai_ss_dance_nyr_p', 'baseline_year_1_arm_1')_1.0	0.044	5.885E-06	5.230
Activity	('sai_ss_dance_nmonth_p', 'baseline_year_1_arm_1')_0.0	-0.076	5.286E-15	14.277
Activity	('sai_ss_dance_nmonth_p', 'baseline_year_1_arm_1')_9.0	0.050	2.396E-07	6.621
Activity	('sai_ss_dance_perwk_p', 'baseline_year_1_arm_1')_0.0	-0.077	3.524E-15	14.453
Activity	('sai_ss_dance_perwk_p', 'baseline_year_1_arm_1')_1.0	0.063	1.055E-10	9.977
Activity	('sai_ss_dance_tspent_p', 'baseline_year_1_arm_1')_0.0	-0.077	1.993E-15	14.700
Activity	('sai_ss_dance_tspent_p', 'baseline_year_1_arm_1')_4.0	0.055	1.912E-08	7.718
Activity	('sai_ss_gym_nyr_p', 'baseline_year_1_arm_1')_0.0	-0.065	1.713E-11	10.766
Activity	('sai_ss_gym_nmonth_p', 'baseline_year_1_arm_1')_0.0	-0.066	1.017E-11	10.993
Activity	('sai_ss_gym_perwk_p', 'baseline_year_1_arm_1')_0.0	-0.066	1.056E-11	10.976
Activity	('sai_ss_gym_perwk_p', 'baseline_year_1_arm_1')_1.0	0.068	3.792E-12	11.421
Activity	('sai_ss_gym_tspent_p', 'baseline_year_1_arm_1')_0.0	-0.065	3.111E-11	10.507
Activity	('sai_ss_gym_tspent_p', 'baseline_year_1_arm_1')_4.0	0.049	3.821E-07	6.418
Activity	('sai_ss_music_nyr_p', 'baseline_year_1_arm_1')_0.0	-0.059	1.115E-09	8.953
Activity	('sai_ss_music_nmonth_p', 'baseline_year_1_arm_1')_0.0	-0.056	7.797E-09	8.108
Activity	('sai_ss_music_perwk_p', 'baseline_year_1_arm_1')_0.0	-0.055	2.003E-08	7.698
Activity	('sai_ss_music_perwk_p', 'baseline_year_1_arm_1')_1.0	0.045	3.411E-06	5.467
Activity	('sai_ss_music_tspent_p', 'baseline_year_1_arm_1')_0.0	-0.056	9.659E-09	8.015
Social Adjustment	('kbi_p_how_well_c_school', 'baseline_year_1_arm_1')_1.0	0.065	3.260E-11	10.487
Social Adjustment	('kbi_p_how_well_c_school', 'baseline_year_1_arm_1')_2.0	-0.050	3.127E-07	6.505
Social Adjustment	('kbi_p_grades_in_school', 'baseline_year_1_arm_1')_1.0	0.058	3.295E-09	8.482
Social Adjustment	('kbi_p_c_spec_serv___4', 'baseline_year_1_arm_1')_0.0	0.046	2.813E-06	5.551
Social Adjustment	('kbi_p_c_spec_serv___4', 'baseline_year_1_arm_1')_1.0	-0.046	2.813E-06	5.551
Substance Use	('tlfb_age_calc_inmonths', 'baseline_year_1_arm_1')	-0.069	1.212E-12	11.917

Substance Use	('hair_status_y', 'baseline_year_1_arm_1')_5.0	-0.047	1.167E-06	5.933
Substance Use	('tlfb_age', 'baseline_year_1_arm_1')_9.0	0.054	3.701E-08	7.432
Substance Use	('tlfb_age', 'baseline_year_1_arm_1')_10.0	-0.052	7.184E-08	7.144
Phys Characteristics	('pds_sex_y', 'baseline_year_1_arm_1')_1.0	-0.151	4.793E-55	54.319
Phys Characteristics	('pds_sex_y', 'baseline_year_1_arm_1')_2.0	0.151	4.793E-55	54.319
Questionnaire	('screen13_y', 'baseline_year_1_arm_1')_0.0	0.063	7.603E-11	10.119
Questionnaire	('scrn_hr_disobey', 'screener_arm_1')_0.0	0.049	3.780E-07	6.423
Questionnaire	('scrn_commondx', 'screener_arm_1')_0.0	0.047	1.096E-06	5.960
Questionnaire	('scrn_commondx', 'screener_arm_1')_1.0	-0.047	1.096E-06	5.960
Diagnostic	('ksads_14_414_p', 'baseline_year_1_arm_1')_0.0	0.053	4.954E-08	7.305
Diagnostic	('ksads_14_414_p', 'baseline_year_1_arm_1')_1.0	-0.053	4.954E-08	7.305
Diagnostic	('ksads_14_419_p', 'baseline_year_1_arm_1')_0.0	0.047	1.280E-06	5.893
Diagnostic	('ksads_14_419_p', 'baseline_year_1_arm_1')_1.0	-0.047	1.280E-06	5.893
Diagnostic	('ksads_14_422_p', 'baseline_year_1_arm_1')_0.0	0.045	4.585E-06	5.339
Diagnostic	('ksads_14_422_p', 'baseline_year_1_arm_1')_1.0	-0.045	4.585E-06	5.339
Diagnostic	('ksads_14_413_p', 'baseline_year_1_arm_1')_0.0	0.058	3.325E-09	8.478
Diagnostic	('ksads_14_413_p', 'baseline_year_1_arm_1')_1.0	-0.058	3.325E-09	8.478
Diagnostic	('ksads_14_420_p', 'baseline_year_1_arm_1')_0.0	0.045	3.100E-06	5.509
Diagnostic	('ksads_14_420_p', 'baseline_year_1_arm_1')_1.0	-0.045	3.100E-06	5.509
Diagnostic	('ksads_14_87_p', 'baseline_year_1_arm_1')_0.0	0.045	3.471E-06	5.460
Diagnostic	('ksads_14_87_p', 'baseline_year_1_arm_1')_1.0	-0.045	3.471E-06	5.460
Diagnostic	('ksads_14_86_p', 'baseline_year_1_arm_1')_0.0	0.044	5.233E-06	5.281
Diagnostic	('ksads_14_86_p', 'baseline_year_1_arm_1')_1.0	-0.044	5.233E-06	5.281
Diagnostic	('ksads_14_412_p', 'baseline_year_1_arm_1')_0.0	0.048	9.031E-07	6.044
Diagnostic	('ksads_14_412_p', 'baseline_year_1_arm_1')_1.0	-0.048	9.031E-07	6.044
Diagnostic	('ksads_14_417_p', 'baseline_year_1_arm_1')_0.0	0.055	1.980E-08	7.703
Diagnostic	('ksads_14_417_p', 'baseline_year_1_arm_1')_1.0	-0.055	1.980E-08	7.703
Diagnostic	('ksads_14_82_p', 'baseline_year_1_arm_1')_0.0	0.047	1.205E-06	5.919
Diagnostic	('ksads_14_82_p', 'baseline_year_1_arm_1')_1.0	-0.047	1.205E-06	5.919
Diagnostic	('ksads_14_83_p', 'baseline_year_1_arm_1')_0.0	0.046	2.349E-06	5.629
Diagnostic	('ksads_14_83_p', 'baseline_year_1_arm_1')_1.0	-0.046	2.349E-06	5.629
Diagnostic	('ksads_14_411_p', 'baseline_year_1_arm_1')_0.0	0.050	3.430E-07	6.465
Diagnostic	('ksads_14_411_p', 'baseline_year_1_arm_1')_1.0	-0.050	3.430E-07	6.465
Diagnostic	('ksads_14_425_p', 'baseline_year_1_arm_1')_0.0	0.047	1.112E-06	5.954
Diagnostic	('ksads_14_425_p', 'baseline_year_1_arm_1')_1.0	-0.047	1.112E-06	5.954

Subtype 3				
**cat_id**	**phe_name**	**r_value**	**p_value**	**-logp_value**
Demographics	('interview_age', 'baseline_year_1_arm_1')	-0.100	6.456E-25	24.190
Demographics	('interview_age', 'screener_arm_1')	-0.100	6.456E-25	24.190
Demographics	('demo_brthdat_v2', 'baseline_year_1_arm_1')	-0.083	1.784E-17	16.749
Demographics	('demo_ed_v2', 'baseline_year_1_arm_1')_3.0	0.063	8.052E-11	10.094
Demographics	('demo_ed_v2', 'baseline_year_1_arm_1')_5.0	-0.076	5.822E-15	14.235
Demographics	('demo_race_a_p___11', 'baseline_year_1_arm_1')_0.0	0.073	6.650E-14	13.177
Demographics	('demo_race_a_p___11', 'baseline_year_1_arm_1')_1.0	-0.073	6.650E-14	13.177
Demographics	('demo_prnt_race_a_v2___11', 'baseline_year_1_arm_1')_0.0	0.066	8.735E-12	11.059
Demographics	('demo_prnt_race_a_v2___11', 'baseline_year_1_arm_1')_1.0	-0.066	8.735E-12	11.059
Socioeconomic	('ethnicity', '')_2.0	-0.064	4.083E-11	10.389
Socioeconomic	('genetic_af_african', 'baseline_year_1_arm_1')	-0.075	1.485E-14	13.828
Socioeconomic	('genetic_af_european', 'baseline_year_1_arm_1')	0.049	4.524E-07	6.344
Social Adjustment	('mex_american15_p', 'baseline_year_1_arm_1')_5.0	-0.044	7.708E-06	5.113
Social Adjustment	('meim_ethnic_id_p', 'baseline_year_1_arm_1')_5.0	-0.059	1.500E-09	8.824
Substance Use	('tlfb_age_calc_inmonths', 'baseline_year_1_arm_1')	-0.099	1.526E-24	23.816
Substance Use	('tlfb_age_month', 'baseline_year_1_arm_1')_11.0	-0.046	1.848E-06	5.733
Substance Use	('tlfb_age', 'baseline_year_1_arm_1')_9.0	0.084	4.084E-18	17.389
Substance Use	('tlfb_age', 'baseline_year_1_arm_1')_10.0	-0.085	2.342E-18	17.630
Phys Exam	('hormone_scr_dhea_rep2', 'baseline_year_1_arm_1')	-0.062	1.459E-10	9.836
Phys Exam	('hormone_scr_ert_mean', 'baseline_year_1_arm_1')	-0.061	3.295E-10	9.482
Phys Exam	('hormone_scr_ert_rep1', 'baseline_year_1_arm_1')	-0.065	2.825E-11	10.549
Phys Exam	('hormone_scr_ert_rep2', 'baseline_year_1_arm_1')	-0.061	4.690E-10	9.329
Phys Characteristics	('pds_2_p', 'baseline_year_1_arm_1')_1.0	0.075	1.758E-14	13.755
Phys Characteristics	('pds_2_p', 'baseline_year_1_arm_1')_3.0	-0.065	1.894E-11	10.723
Phys Characteristics	('pds_3_p', 'baseline_year_1_arm_1')_1.0	0.067	7.796E-12	11.108
Phys Characteristics	('pds_3_p', 'baseline_year_1_arm_1')_3.0	-0.054	2.208E-08	7.656
**Subtype 4**				
**cat_id**	**phe_name**	**r_value**	**p_value**	**-logp_value**
Demographics	('interview_age', 'baseline_year_1_arm_1')	-0.052	8.234E-08	7.084
Demographics	('interview_age', 'screener_arm_1')	-0.052	8.234E-08	7.084
Demographics	('demo_race_a_p___10', 'baseline_year_1_arm_1')_0.0	-0.048	7.511E-07	6.124
Demographics	('demo_race_a_p___10', 'baseline_year_1_arm_1')_1.0	0.048	7.511E-07	6.124
Demographics	('demo_race_a_p___25', 'baseline_year_1_arm_1')_0.0	0.044	6.526E-06	5.185
Demographics	('demo_race_a_p___25', 'baseline_year_1_arm_1')_1.0	-0.044	6.526E-06	5.185
Demographics	('demo_ethn_v2', 'baseline_year_1_arm_1')_1.0	-0.056	1.110E-08	7.955
Demographics	('demo_ethn_v2', 'baseline_year_1_arm_1')_2.0	0.056	1.110E-08	7.955

Demographics	('demo_prnt_race_a_v2___10', 'baseline_year_1_arm_1')_0.0	-0.047	1.607E-06	5.794
Demographics	('demo_prnt_race_a_v2___10', 'baseline_year_1_arm_1')_1.0	0.047	1.607E-06	5.794
Demographics	('demo_prnt_ethn_v2', 'baseline_year_1_arm_1')_1.0	-0.048	7.135E-07	6.147
Demographics	('demo_prnt_ethn_v2', 'baseline_year_1_arm_1')_2.0	0.048	7.135E-07	6.147
Socioeconomic	('ethnicity', '')_1.0	0.065	2.272E-11	10.644
Socioeconomic	('ethnicity', '')_3.0	-0.056	8.124E-09	8.090
Socioeconomic	('genetic_af_european', 'baseline_year_1_arm_1')	0.066	8.599E-12	11.066
Socioeconomic	('genetic_af_american', 'baseline_year_1_arm_1')	-0.081	1.196E-16	15.922
Socioeconomic	('reshist_addr1_adi_edu_h', 'baseline_year_1_arm_1')	0.045	4.605E-06	5.337
Socioeconomic	('reshist_addr1_svi_sin_20142018', 'baseline_year_1_arm_1')	-0.047	1.321E-06	5.879
Cognitive	('pea_wiscv_trs', 'baseline_year_1_arm_1')	0.056	6.748E-09	8.171
Cognitive	('pea_wiscv_tss', 'baseline_year_1_arm_1')	0.062	1.517E-10	9.819
Behavior	('cbcl_q53_p', 'baseline_year_1_arm_1')_0.0	0.053	4.500E-08	7.347
Behavior	('cbcl_q55_p', 'baseline_year_1_arm_1')_0.0	0.082	4.024E-17	16.395
Behavior	('cbcl_q55_p', 'baseline_year_1_arm_1')_1.0	-0.053	4.073E-08	7.390
Behavior	('cbcl_q55_p', 'baseline_year_1_arm_1')_2.0	-0.064	3.723E-11	10.429
Behavior	('cbcl_q102_p', 'baseline_year_1_arm_1')_0.0	0.045	4.743E-06	5.324
Behavior	('cbcl_q102_p', 'baseline_year_1_arm_1')_1.0	-0.045	4.174E-06	5.379
Activity	('sai_p_activities___6', 'baseline_year_1_arm_1')_0.0	-0.043	7.859E-06	5.105
Activity	('sai_p_activities___6', 'baseline_year_1_arm_1')_1.0	0.043	7.859E-06	5.105
Activity	('sai_ss_gym_nmonth_p', 'baseline_year_1_arm_1')_0.0	-0.044	5.324E-06	5.274
Activity	('sai_ss_gym_perwk_p', 'baseline_year_1_arm_1')_1.0	0.050	2.383E-07	6.623
Social Adjustment	('kbi_p_how_well_c_school', 'baseline_year_1_arm_1')_1.0	0.046	2.267E-06	5.645
Social Adjustment	('kbi_p_how_well_c_school', 'baseline_year_1_arm_1')_3.0	-0.049	4.817E-07	6.317
Social Adjustment	('kbi_p_grades_in_school', 'baseline_year_1_arm_1')_1.0	0.044	6.791E-06	5.168
Social Adjustment	('kbi_p_c_spec_serv___4', 'baseline_year_1_arm_1')_0.0	0.048	8.740E-07	6.058
Social Adjustment	('kbi_p_c_spec_serv___4', 'baseline_year_1_arm_1')_1.0	-0.048	8.740E-07	6.058
Social Adjustment	('meim_ethnic_id_p', 'baseline_year_1_arm_1')_1.0	0.046	2.343E-06	5.630
Social Adjustment	('meim_ethnic_id_p', 'baseline_year_1_arm_1')_4.0	-0.048	1.032E-06	5.986
Substance Use	('tlfb_age_calc_inmonths', 'baseline_year_1_arm_1')	-0.050	2.919E-07	6.535
Questionnaire	('bisbas17_y', 'baseline_year_1_arm_1')_1.0	0.045	4.605E-06	5.337
Questionnaire	('screen2_wkdy_y', 'baseline_year_1_arm_1')_0.0	0.046	2.929E-06	5.533
Questionnaire	('screen8_wknd_y', 'baseline_year_1_arm_1')_0.0	0.044	5.166E-06	5.287
		**Subtype 5**		
**cat_id**	**phe_name**	**r_value**	**p_value**	**-logp_value**
Demographics	('sex_official', '')_F	-0.165	1.784E-65	64.749
Demographics	('sex_official', '')_M	0.165	1.784E-65	64.749
Demographics	('demo_sex_v2', 'baseline_year_1_arm_1')_1.0	0.165	3.271E-65	64.485

Demographics	('demo_sex_v2', 'baseline_year_1_arm_1')_2.0	-0.165	3.706E-65	64.431
Demographics	('demo_gender_id_v2', 'baseline_year_1_arm_1')_1.0	0.164	1.242E-64	63.906
Demographics	('demo_gender_id_v2', 'baseline_year_1_arm_1')_2.0	-0.165	1.634E-65	64.787
Demographics	('demo_race_a_p___11', 'baseline_year_1_arm_1')_0.0	0.107	4.838E-28	27.315
Demographics	('demo_race_a_p___11', 'baseline_year_1_arm_1')_1.0	-0.107	4.838E-28	27.315
Demographics	('demo_prnt_race_a_v2___11', 'baseline_year_1_arm_1')_0.0	0.089	4.990E-20	19.302
Demographics	('demo_prnt_race_a_v2___11', 'baseline_year_1_arm_1')_1.0	-0.089	4.990E-20	19.302
Socioeconomic	('ethnicity', '')_2.0	-0.083	1.032E-17	16.986
Socioeconomic	('ethnicity', '')_3.0	0.043	7.948E-06	5.100
Socioeconomic	('genetic_af_african', 'baseline_year_1_arm_1')	-0.103	3.991E-26	25.399
Socioeconomic	('genetic_af_european', 'baseline_year_1_arm_1')	0.051	2.047E-07	6.689
Socioeconomic	('genetic_af_american', 'baseline_year_1_arm_1')	0.061	2.625E-10	9.581
Activity	('sai_p_activities___0', 'baseline_year_1_arm_1')_0.0	0.110	7.986E-30	29.098
Activity	('sai_p_activities___0', 'baseline_year_1_arm_1')_1.0	-0.110	7.986E-30	29.098
Activity	('sai_p_activities___1', 'baseline_year_1_arm_1')_0.0	-0.056	6.495E-09	8.187
Activity	('sai_p_activities___1', 'baseline_year_1_arm_1')_1.0	0.056	6.495E-09	8.187
Activity	('sai_p_activities___6', 'baseline_year_1_arm_1')_0.0	0.058	1.916E-09	8.717
Activity	('sai_p_activities___6', 'baseline_year_1_arm_1')_1.0	-0.058	1.916E-09	8.717
Activity	('sai_p_activities___10', 'baseline_year_1_arm_1')_0.0	-0.049	4.718E-07	6.326
Activity	('sai_p_activities___10', 'baseline_year_1_arm_1')_1.0	0.049	4.718E-07	6.326
Activity	('sai_ss_dance_nyr_p', 'baseline_year_1_arm_1')_0.0	0.110	8.279E-30	29.082
Activity	('sai_ss_dance_nyr_p', 'baseline_year_1_arm_1')_1.0	-0.065	2.211E-11	10.655
Activity	('sai_ss_dance_nyr_p', 'baseline_year_1_arm_1')_2.0	-0.051	1.382E-07	6.859
Activity	('sai_ss_dance_nmonth_p', 'baseline_year_1_arm_1')_0.0	0.109	4.934E-29	28.307
Activity	('sai_ss_dance_nmonth_p', 'baseline_year_1_arm_1')_9.0	-0.046	2.523E-06	5.598
Activity	('sai_ss_dance_perwk_p', 'baseline_year_1_arm_1')_0.0	0.111	4.035E-30	29.394
Activity	('sai_ss_dance_perwk_p', 'baseline_year_1_arm_1')_1.0	-0.072	1.483E-13	12.829
Activity	('sai_ss_dance_perwk_p', 'baseline_year_1_arm_1')_2.0	-0.059	1.733E-09	8.761
Activity	('sai_ss_dance_tspent_p', 'baseline_year_1_arm_1')_0.0	0.110	9.080E-30	29.042
Activity	('sai_ss_dance_tspent_p', 'baseline_year_1_arm_1')_3.0	-0.046	2.002E-06	5.699
Activity	('sai_ss_dance_tspent_p', 'baseline_year_1_arm_1')_4.0	-0.063	1.186E-10	9.926
Activity	('sai_ss_dance_tspent_p', 'baseline_year_1_arm_1')_5.0	-0.044	7.343E-06	5.134
Activity	('sai_ss_base_nyr_p', 'baseline_year_1_arm_1')_0.0	-0.057	4.490E-09	8.348
Activity	('sai_ss_base_nyr_p', 'baseline_year_1_arm_1')_4.0	0.046	2.914E-06	5.535
Activity	('sai_ss_base_nmonth_p', 'baseline_year_1_arm_1')_0.0	-0.057	5.412E-09	8.267
Activity	('sai_ss_base_perwk_p', 'baseline_year_1_arm_1')_0.0	-0.057	3.749E-09	8.426

Activity	('sai_ss_base_tspent_p', 'baseline_year_1_arm_1')_0.0	-0.058	3.094E-09	8.510
Activity	('sai_ss_gym_nyr_p', 'baseline_year_1_arm_1')_0.0	0.059	1.752E-09	8.757
Activity	('sai_ss_gym_nmonth_p', 'baseline_year_1_arm_1')_0.0	0.059	9.840E-10	9.007
Activity	('sai_ss_gym_perwk_p', 'baseline_year_1_arm_1')_0.0	0.058	1.943E-09	8.711
Activity	('sai_ss_gym_perwk_p', 'baseline_year_1_arm_1')_1.0	-0.050	3.246E-07	6.489
Activity	('sai_ss_gym_tspent_p', 'baseline_year_1_arm_1')_0.0	0.058	1.846E-09	8.734
Activity	('sai_ss_m_arts_nyr_p', 'baseline_year_1_arm_1')_0.0	-0.049	4.197E-07	6.377
Activity	('sai_ss_m_arts_nmonth_p', 'baseline_year_1_arm_1')_0.0	-0.045	3.724E-06	5.429
Activity	('sai_ss_m_arts_perwk_p', 'baseline_year_1_arm_1')_0.0	-0.048	6.526E-07	6.185
Activity	('sai_ss_m_arts_perwk_p', 'baseline_year_1_arm_1')_2.0	0.051	1.316E-07	6.881
Activity	('sai_ss_m_arts_tspent_p', 'baseline_year_1_arm_1')_0.0	-0.048	9.892E-07	6.005
Social Adjustment	('pmq_y_ss_mean', 'baseline_year_1_arm_1')	-0.049	5.819E-07	6.235
Social Adjustment	('meim_ethnic_id_p', 'baseline_year_1_arm_1')_5.0	-0.075	1.724E-14	13.763
Phys Exam	('hormone_scr_dhea_rep2', 'baseline_year_1_arm_1')	-0.044	6.249E-06	5.204
Phys Characteristics	('pds_sex_y', 'baseline_year_1_arm_1')_1.0	0.163	6.979E-64	63.156
Phys Characteristics	('pds_sex_y', 'baseline_year_1_arm_1')_2.0	-0.163	6.979E-64	63.156
Phys Characteristics	('pds_2_p', 'baseline_year_1_arm_1')_1.0	0.069	9.163E-13	12.038
Phys Characteristics	('pds_2_p', 'baseline_year_1_arm_1')_3.0	-0.061	3.961E-10	9.402
Phys Characteristics	('pds_3_p', 'baseline_year_1_arm_1')_1.0	0.072	1.620E-13	12.790
Phys Characteristics	('pds_3_p', 'baseline_year_1_arm_1')_2.0	-0.047	1.725E-06	5.763
Phys Characteristics	('pds_3_p', 'baseline_year_1_arm_1')_3.0	-0.047	1.181E-06	5.928
Questionnaire	('screen13_y', 'baseline_year_1_arm_1')_0.0	-0.054	2.448E-08	7.611
Questionnaire	('screen10_wknd_y', 'baseline_year_1_arm_1')_0.0	0.053	5.928E-08	7.227
