# Supplementary material for: Latent brain subtypes of chronotype reveal unique behavioral and health profiles across population cohorts
Source: Nat Commun. 2025 Dec 22;16:11550. doi: 10.1038/s41467-025-66784-8 (PMC12749713; doi:10.1038/s41467-025-66784-8)
Supplement: Supplementary file 2 — Reporting Summary [file 41467_2025_66784_MOESM2_ESM.pdf]

nature portfolio

Corresponding author(s): Danilo Bzdok

Last updated by author(s): Nov 4, 2025

Reporting Summary

Nature Portfolio wishes to improve the reproducibility of the work that we publish. This form provides structure for consistency and transparency in reporting. For further information on Nature Portfolio policies, see our Editorial Policies and the Editorial Policy Checklist.

Statistics

For all statistical analyses, confirm that the following items are present in the figure legend, table legend, main text, or Methods section.

n/a	Confirmed	
☐	☒	The exact sample size (*n*) for each experimental group/condition, given as a discrete number and unit of measurement
☐	☒	A statement on whether measurements were taken from distinct samples or whether the same sample was measured repeatedly
☐	☒	The statistical test(s) used AND whether they are one- or two-sided<br>*Only common tests should be described solely by name; describe more complex techniques in the Methods section.*
☐	☒	A description of all covariates tested
☐	☒	A description of any assumptions or corrections, such as tests of normality and adjustment for multiple comparisons
☐	☒	A full description of the statistical parameters including central tendency (e.g. means) or other basic estimates (e.g. regression coefficient) AND variation (e.g. standard deviation) or associated estimates of uncertainty (e.g. confidence intervals)
☐	☒	For null hypothesis testing, the test statistic (e.g. *F*, *t*, *r*) with confidence intervals, effect sizes, degrees of freedom and *P* value noted<br>*Give P values as exact values whenever suitable.*
☒	☐	For Bayesian analysis, information on the choice of priors and Markov chain Monte Carlo settings
☒	☐	For hierarchical and complex designs, identification of the appropriate level for tests and full reporting of outcomes
☐	☒	Estimates of effect sizes (e.g. Cohen's *d*, Pearson's *r*), indicating how they were calculated

Our web collection on statistics for biologists contains articles on many of the points above.

Software and code

Policy information about availability of computer code

Data collection	No data were collected for this study.
Data analysis	The processing scripts for this work were implemented in Python (3.9.18) and utilized the following packages: scikit-learn (1.5.2), numpy (1.26.4), pandas (2.2.3), scipy (1.13.1), nilearn (0.11.0), mateplotlib (3.8.4), joblib (1.4.2), fMRIPrep 21.02, Nipype 1.6.1, ANTs 2.3.3, FSL 6.0.5.1, FreeSurfer 6.0.1. All related scripts are publicly accessible and archived at https://doi.org/10.5281/zenodo.17524317 .

For manuscripts utilizing custom algorithms or software that are central to the research but not yet described in published literature, software must be made available to editors and reviewers. We strongly encourage code deposition in a community repository (e.g. GitHub). See the Nature Portfolio guidelines for submitting code & software for further information.

Data

Policy information about availability of data

All manuscripts must include a data availability statement. This statement should provide the following information, where applicable:

- Accession codes, unique identifiers, or web links for publicly available datasets
- A description of any restrictions on data availability
- For clinical datasets or third party data, please ensure that the statement adheres to our policy

The UK Biobank data are available to other investigators online (https://www.ukbiobank.ac.uk/). The ABCD Study® data are available to other investigators online (https://abcdstudy.org/). The Harvard–Oxford atlas, Probabilistic cerebellar atlas, and Johns Hopkins University atlas are accessible online (https://fsl.fmrib.ox.ac.uk/fsl/fslwiki/Atlases). Source Data are provided with this paper.

Research involving human participants, their data, or biological material

Policy information about studies with human participants or human data. See also policy information about sex, gender (identity/presentation), and sexual orientation and race, ethnicity and racism.

Reporting on sex and gender	We included 27,030 UK Biobank participants with three brain modalities (structural MRI, diffusion MRI, resting-state fMRI). Among these participants, 54% were females. Moreover, we included 10,550 children from the ABCD study (48.04% females). Both of them were self-reported data.
Reporting on race, ethnicity, or other socially relevant groupings	Ethnicity, Education level, employment status, and other socially relevant groupings (e.g., household, attendance/disability/mobility allowance) were among the sociodemographic factors considered in the phenome-wide analysis.
Population characteristics	We included 27,030 UK Biobank participants with three brain modalities (structural MRI, diffusion MRI, resting-state fMRI). Participants were recruited between the ages of 40 and 70 years (mean age with standard deviation: 55.37 ± 7.39), 54% were females. Moreover, we included 10,550 children from the ABCD study (mean age +/- SD: 119.01 +/- 7.51 months, 48.04% females).
Recruitment	Recruitment was done as part of the UKbiobank initiative by flyers and other means common in epidemiological research. For details on representativeness see Frey et al., 2017. Details on recruitment for the ABCD study can be found in Garavan et al., 2018.
Ethics oversight	UK Biobank participants gave written, informed consent for the study, which was approved by the Research Ethics Committee. The present analyses were conducted under UK Biobank application number 25163. Further information on the consent procedure can be found here (biobank.ctsu.ox.ac.uk/crystal/field.cgi?id=200).<br>All protocols for the ABCD Study are approved by a central Institutional Review Board (cIRB) at the University of California, San Diego, for the ethical review and approval of the research protocol, with a few sites obtaining local IRB approval (https://www.sciencedirect.com/science/article/pii/S1878929317302268#sec0040). Caregivers provided written, informed consent, and children gave verbal assent to all research protocols, in accordance with U.S. Department of Health and Human Services (HHS) regulations.

Note that full information on the approval of the study protocol must also be provided in the manuscript.

Field-specific reporting

Please select the one below that is the best fit for your research. If you are not sure, read the appropriate sections before making your selection.

☒ Life sciences ☐ Behavioural & social sciences ☐ Ecological, evolutionary & environmental sciences

For a reference copy of the document with all sections, see nature.com/documents/nr-reporting-summary-flat.pdf

Life sciences study design

All studies must disclose on these points even when the disclosure is negative.

Sample size	we included 27,030 UK Biobank particpants and 10,550 subjects from ABCD study.
Data exclusions	For UK Biobank, subjects displaying disparities in chronotype assessment between the initial visit and the first brain-imaging visit were excluded. Furthermore, to avoid contamination by effects due to shift work, individuals with a history of shift work (defined as jobs involving shift work with data-fields 826-0.0 and 826-2.0, and jobs involving night shift work with data-fields 3426-0.0 and 3426-2.0) were excluded. Our study hence ultimately included 27,030 subjects with neuroimaging measures, covering i) gray matter morphology (T1-weighted MRI, sMRI), ii) white matter tracts (diffusion MRI, dMRI), and iii) functional connectivity (resting-state fMRI, rs-fMRI).<br>For the ABCD study, gray matter volume was successfully derived for 10,550 subjects using the fMRIPrep pipeline, without the application of specific exclusion criteria.
Replication	We implemented a non-parametric permutation procedure to evaluate the statistical robustness of the dominant partial least squares (PLS) components. After 1,000 permutations, five components were statistically significant at a threshold of $P < 0.001$. To quantify the relative importance of each brain feature for the significant PLS components, we applied a bootstrap resampling strategy to the PLS model to obtain the distribution of brain feature loadings. After 1,000 bootstrap iterations, we derived the distributions of brain loadings for all significant components, using a two-sided 5–95% confidence interval. All replication attempts were successful.
Randomization	Randomization was not relevant to our study as there were no experimental manipulations. Participants did not receive any treatment.
Blinding	Blinding was not relevant to our study. Blinding is withholding information about treatment assignment. However, there was no treatment or experimental manipulation in our study.

Reporting for specific materials, systems and methods

We require information from authors about some types of materials, experimental systems and methods used in many studies. Here, indicate whether each material, system or method listed is relevant to your study. If you are not sure if a list item applies to your research, read the appropriate section before selecting a response.

Materials & experimental systems

n/a	Involved in the study
☒	☐ Antibodies
☒	☐ Eukaryotic cell lines
☒	☐ Palaeontology and archaeology
☒	☐ Animals and other organisms
☒	☐ Clinical data
☒	☐ Dual use research of concern
☒	☐ Plants

Methods

n/a	Involved in the study
☒	☐ ChIP-seq
☒	☐ Flow cytometry
☐	☒ MRI-based neuroimaging

Plants

Seed stocks: N/A

Novel plant genotypes: N/A

Authentication: N/A

Magnetic resonance imaging

Experimental design

Design type: Our data include resting-state fMRI.

Design specifications: There is an experimental unit for the resting-state fMRI scan. The resting-state fMRI scan is continuous, with no intervals between trials or blocks within the scan itself.
Duration: 6 minutes (490 timepoints)
TR: 0.735 s
TE: 39ms
The full protocol PDF is provided at: https://biobank.ctsu.ox.ac.uk/crystal/refer.cgi?id=2367

Behavioral performance measures: Behavioral performance in the MRI scanner was not used in this study.

Acquisition

Imaging type(s): Our data include structural MRI (T1), diffusion MRI, and resting-state fMRI.

Field strength: 3T

Sequence & imaging parameters: The ABCD study MRI data acquisition for the structural covers several pages of full detail, which is fully provided previously in Hagler et al., 2019.
For UK Biobank: MRI data acquisition for the structural and functional modalities covers several pages of full detail, which is fully provided previously in references (Miller et al. 2016 and Alfaro-Almagro et al. 2018.). The full PDf of UK Biobank brain imaging documentation is also provided at: https://biobank.ctsu.ox.ac.uk/crystal/crystal/docs/brain_mri.pdf

Area of acquisition: Siemens' auto-align was used to include the full brain in the imaged field-of-view; this was checked (and corrected if necessary) by the radiographer.

Diffusion MRI ☒ Used ☐ Not used

Parameters: Please see above for information about full details.

Preprocessing

Preprocessing software: For UK Biobank, see above (covered previously in full detail in Miller et al. 2016 and Alfaro-Almagro et al. 2018). The full PDF of UK Biobank brain imaging documentation is provided at: https://biobank.ctsu.ox.ac.uk/crystal/crystal/docs/brain_mri.pdf.
For the ABCD study, MRI data were preprocessed by using fMRIPrep v21.0.2.

Normalization: For UK Biobank, see bove (covered previously in full detail in Miller et al. 2016 and Alfaro-Almagro et al. 2018). The full PDf of UK Biobank brain imaging documentation is provided at: https://biobank.ctsu.ox.ac.uk/crystal/crystal/docs/brain_mri.pdf.

	For the ABCD study, volume-based spatial normalization to two standard spaces (MNI152NLin2009cAsym, MNI152NLin6Asym) was performed through nonlinear registration with antsRegistration (ANTs 2.3.3), using brain-extracted versions of both T1w reference and the T1w template. The following templates were selected for spatial normalization: ICBM 152 Nonlinear Asymmetrical template version 2009c, FSL's MNI ICBM 152 non-linear 6th Generation Asymmetric Average Brain Stereotaxic Registration Model.
Normalization template	MNI152
Noise and artifact removal	For UK Biobank, see above (covered previously in full detail in Miller et al. 2016 and Alfaro-Almagro et al. 2018). The full PDf of UK Biobank brain imaging documentation is provided at: https://biobank.ctsu.ox.ac.uk/crystal/crystal/docs/brain_mri.pdf Moreover, before downstream analysis, the nuisance variables that could potentially contaminate the interindividual brain variation of interest were regressed out: these variables outside primary scientific interest included body mass index, head size, head motion during task-related brain scans, head motion during resting-state fMRI scanning, head position and receiver coil in the scanner (x, y, and z), position of the scanner table, as well as the date and acquisition site. Similarly, for the ABCD study, we regressed out the total intracranial volume, scanning sites, and sleep phenotypes (elements name: from "sleepdisturb1_p" to "sleepdisturb26_p"), as these could account for interindividual variations in volume that might confound the primary focus of the study.
Volume censoring	For UK Biobank, see above (covered previously in full detail in Miller et al. 2016 and Alfaro-Almagro et al. 2018). The full PDf of UK Biobank brain imaging documentation is provided at: https://biobank.ctsu.ox.ac.uk/crystal/crystal/docs/brain_mri.pdf. For the ABCD study, MRI data were preprocessed by using fMRIPrep v21.0.2.

Statistical modeling & inference

Model type and settings	N/A
Effect(s) tested	N/A

Specify type of analysis: ☐ Whole brain ☒ ROI-based ☐ Both

Anatomical location(s): ROIs were generated based on Harvard-Oxford atlas (111 regions from T1-weighted structural MRI); Diedrichsen cerebellar atlas (25 ROIs based on T1-weighted MRI) and Johns Hopkins University Atlas (48 ROIs derived from diffusion-weighted MRI). Atlas are accessible online https://fsl.fmrib.ox.ac.uk/fsl/fslwiki/Atlases

Statistic type for inference (See Eklund et al. 2016)	This study does not include task fMRI, and inference was not carried out based on image-derived phenotypes (IDPs) derived from the whole brain. For details on obtaining the ROI, please see above (covered previously in full detail in Miller et al. 2016 and Alfaro-Almagro et al. 2018). The full PDf of UK Biobank brain imaging documentation is provided at: https://biobank.ctsu.ox.ac.uk/crystal/crystal/docs/brain_mri.pdf
Correction	See above.

Models & analysis

n/a	Involved in the study
☐	☒ Functional and/or effective connectivity
☒	☐ Graph analysis
☐	☒ Multivariate modeling or predictive analysis

Functional and/or effective connectivity	The resting-state functional connectivity (partial correlation) based on 21 brain components of spatiotemporally coherent networks derived from group ICA analysis (see Kernbach et al., 2018).
Multivariate modeling and predictive analysis	We used partial least squares (PLS) as a supervised pattern-learning approach to identify patterns associated with both brain data and chronotype. The resulting dominant PLS components were evaluated for statistical robustness using 1,000 non-parametric permutations.
